# To senesce or not to senesce: how primary human fibroblasts decide their cell fate after DNA damage

**DOI:** 10.18632/aging.100883

**Published:** 2016-01-30

**Authors:** Gabriel Kollarovic, Maja Studencka, Lyubomira Ivanova, Claudia Lauenstein, Kristina Heinze, Anastasiya Lapytsko, Soheil Rastgou Talemi, Ana Sofia Figueiredo, Jörg Schaber

**Affiliations:** ^1^ Institute for Experimental Internal Medicine, Medical Faculty, Otto-von-Guericke University, Magdeburg, Germany; ^2^ Cancer Research Institute, Slovak Academy of Sciences, Bratislava, Slovakia

**Keywords:** G1-S transition, bistable hysteresis switch, dynamic model, Cdk2/p21 ratio, cumulative DNA damage

## Abstract

Excessive DNA damage can induce an irreversible cell cycle arrest, called senescence, which is generally perceived as an important tumour-suppressor mechanism. However, it is unclear how cells decide whether to senesce or not after DNA damage. By combining experimental data with a parameterized mathematical model we elucidate this cell fate decision at the G1-S transition. Our model provides a quantitative and conceptually new understanding of how human fibroblasts decide whether DNA damage is beyond repair and senesce. Model and data imply that the G1-S transition is regulated by a bistable hysteresis switch with respect to Cdk2 activity, which in turn is controlled by the Cdk2/p21 ratio rather than cyclin abundance. We experimentally confirm the resulting predictions that to induce senescence i) in healthy cells both high initial and elevated background DNA damage are necessary and sufficient, and ii) in already damaged cells much lower additional DNA damage is sufficient. Our study provides a mechanistic explanation of a) how noise in protein abundances allows cells to overcome the G1-S arrest even with substantial DNA damage, potentially leading to neoplasia, and b) how accumulating DNA damage with age increasingly sensitizes cells for senescence.

## INTRODUCTION

It is well known that upon excessive DNA damage several cell types including primary human fibroblasts permanently and irreversibly arrest their cell cycle and develop a specific phenotype called senescence. Senescence is characterized by a specific morphology (vacuolated and enlarged cells) and gene expression pattern, and is generally perceived as an important tumor-suppressor mechanism [1-4]. Commonly accepted markers of senescence, in conjunction with swift cell cycle arrest, are increased senescence-associated β-galactosidase activity (SA-βG) and up-regulation of cyclin-dependent kinase (CDK) inhibitors, like p21 and p16 [5]. However, this phenotype develops over a period of about one week [6, 7], even though DNA damage is largely repaired within one or two days [8]. Therefore, the cell fate decision, whether DNA damage is beyond repair or not and whether to senesce or not to senesce, must be taken shortly after DNA damage occurred.

Literature suggested that the cell fate decision between permanent arrest, i.e. senescence, and transient arrest is mediated by a permanent DNA damage signal emanating from unrepairable telomeric DNA damage [9-13]. Double-strand breaks in the telomeres cannot be repaired, a mechanism that usually prevents chromosome fusions [14]. Consequently, the number of persistent telomere-associated DNA damage increases with increasing irradiation dose [9, 10]. The permanent cell cycle arrest is then presumably mediated by a permanently elevated background DNA damage signal, which halts the cell cycle through well-known pathways, e.g., the p53-p21 pathway and the p16-Rb-E2F pathway. Both pathways have been extensively reviewed [15-20] and are shortly described in Supplemental Data 1 (Figure S1).

**Figure 1:**
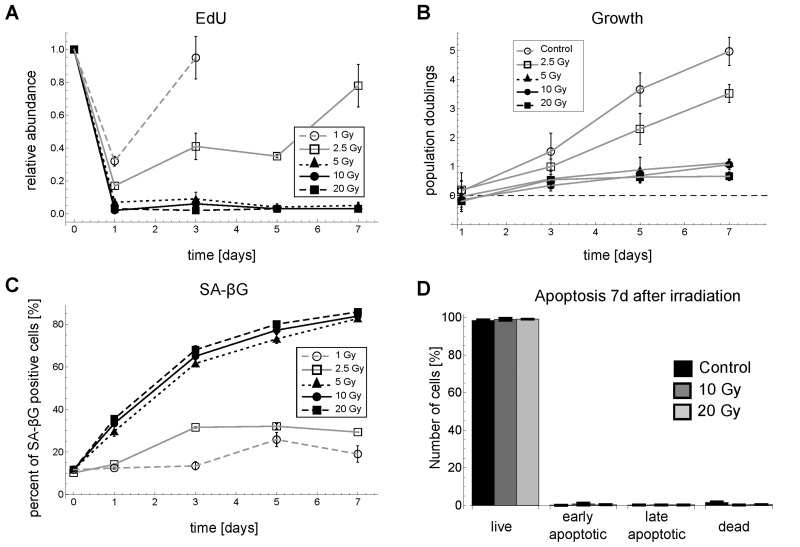
Time series for G1-S arrest and senescence for different γ-irradiation regimes in MRC5 fibroblasts. (**A**) Relative abundance of EdU positive cells (mean ± SEM (n≥3)). (**B**) Population doublings (mean ± SEM for at least three independent cell counts with >100 cells each) (**C**) SA-βG activity (mean ± SEM (n=3)). (**D**) Percentage of live cells (negative for both Annexin V and propidium iodide (PI), early apoptotic cells (positive for Annexin V and negative for PI), late apoptotic/necrotic cells (positive for both Annexin V and PI) and dead cells (negative for Annexin V and positive for PI (mean ± SE (n=3)). Representative FACS scatter plots for EdU and SA-βG measurements are shown in Figure S5.

The hypothesis that a permanently elevated p21-mediated background DNA damage signal is sufficient to arrest the cell cycle and induce senescence, is evidenced by the fact that ionizing radiation (IR)-induced senescence can partly be rescued by repression of p21 [21]. However, the transient increase in double-strand breaks after 2.5 Gy IR drastically exceeds the number of persistent telomeric double-strand breaks after 20 Gy IR [8-10]. Yet, 2.5 Gy IR only transiently arrests cells, whereas 20 Gy IR leads to senescence (Figure 1). Moreover, the difference in persistent DNA damage between 2.5 and 20 Gy IR is merely around four double-strand breaks, a number which is almost within the background damage of around two double-strand breaks, measured by γH2AX foci [10].

Here we examine how cells discriminate between high induced and slightly elevated background DNA damage in order to take a cell fate decision. Specifically, we focus on the G1-S transition. To this end, we used published dynamics of DNA double-strand breaks after different doses of IR and measured corresponding dynamics of well-known players that mediate DNA damage signaling and G1-S arrest for MRC5 primary human diploid fibroblasts. We developed several literature-based mathematical models, which can quantitatively recapitulate our measured data. We selected the model that was best supported by the data in terms of parsimony. The ensuing parameterized model suggests that G1-S arrest is regulated by a robust bistable hysteresis-switch, whose bistable region, i.e. the region where the cell fate decision between proliferation and senescence is taken, is between six and 12 double-strand breaks. Thus, the model provides a mechanistic and quantitative explanation of how cells count double-strand breaks and decide whether the non-repairable DNA damage is too much to proliferate. Both high initial and elevated background DNA damage are necessary and sufficient to induce a permanent cell cycle exit at the G1-S transition. Accordingly, the model predicted that i) repeated low dose irradiation increases permanent DNA damage, but fails to induce cell cycle arrest and senescence, and ii) in already damaged cells a much lower additional dose of IR is sufficient to induce senescence. Both predictions were quantitatively corroborated by dedicated follow-up experiments. The model also suggests that, opposed to the commonly accepted opinion of a cyclin abundance-regulated G1-S transition, the Cdk2/p21 ratio controls Cdk2 activity and DNA damage induced G1-S arrest. We provide evidence that this seems to be a common mechanism for several primary human fibroblasts.

Our model constitutes the first step towards a fully parameterized model of the human DNA damage regulated cell cycle. Such quantitative models hold the key for conceptually new insights into human cell cycle regulation, which in turn are relevant for clinical applications.

## RESULTS

### Experimental analysis of the ionizing radiation (IR) induced G1-S arrest of MRC5 human fibroblasts

#### The cell fate decision whether to senesce or not is taken between 2.5 and 10 Gy IR

Markers of cell cycle progression and senescence like EdU incorporation rate, a marker for DNA synthesis, population doublings, and β-galactosidase activity (SA-βG) reveal a clear cell fate switch between 2.5 and 5 Gy IR for MRC5 primary human fibroblasts (Figure 1). The EdU incorporation rates as well as cell numbers revealed that for doses lower than 5 Gy the G1-S arrest is only transient, where the length of arrest increases with irradiation dose. For doses above 2.5 Gy the arrest lasted for at least one week (Figure 1A,B). SA-βG changed significantly over one week only for irradiation levels above 5 Gy (Figure 1C). These results indicated that for doses ≥5 Gy IR cells go to senescence, i.e. permanently arrest the cell cycle. Thus, we chose 2.5 and 10 Gy as two representative irradiation regimes, which lead to different cell fates, i.e. transient arrest and senescence, after IR-induced DNA damage. We did not observe cell death for up to 20 Gy IR, evidenced by cell count and Annexin V assay (Figure 1D). This is in line with earlier reports [22, 23].

#### MRC5 fibroblasts arrest in both G1 as well as G2 phase

Recently, it has been reported that mainly, if not exclusively, tetraploid cells (4n cells) senesce [24-26]. In our study, both diploid and tetraploid MRC5 cells senesced, as evidenced by increased SA-βG activity and basically constant DNA content distributions (Figure S5, Supplementary Figures). In fact, most of the cells (75%) arrested in G1 phase making it convenient to study this phase also by population-based measures such as Western blots.

#### Immediate G1-S arrest in MRC5 human primary fibroblasts is not regulated by the p16-Rb-E2F pathway or Cdc25A

**Figure 2:**
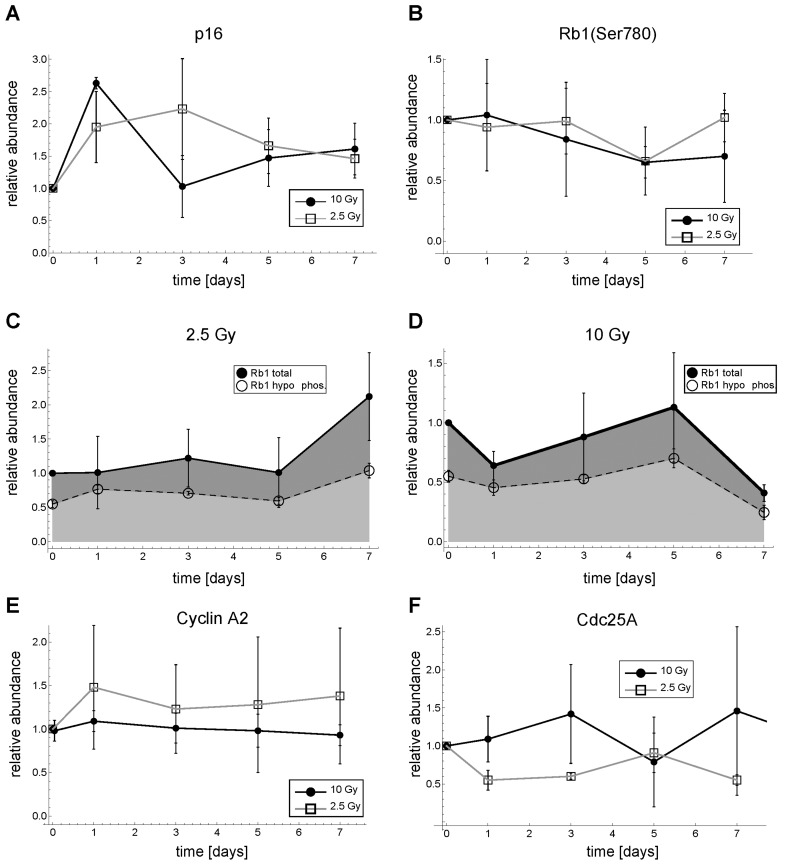
Dynamics of p16, Rb, Cyclin A2 and Cdc25A expression after γ-irradiation in MRC5 human fibroblasts. (**A**) Relative p16 abundance. (**B**) Relative Rb1(Ser780) abundance (**C**) Total and hypo-phosphorylated Rb1 protein for 2.5 Gy IR. (**D**) Total and hypo-phosphorylated Rb1 protein for 10 Gy IR. (**E**) Relative Cyclin A2 abundance (**F**) Relative Cdc25A abundance. Error bars indicate SEM (n≥3). Representative Western Blots are shown in Figure S6, Supplemental Figures.

**Figure 3:**
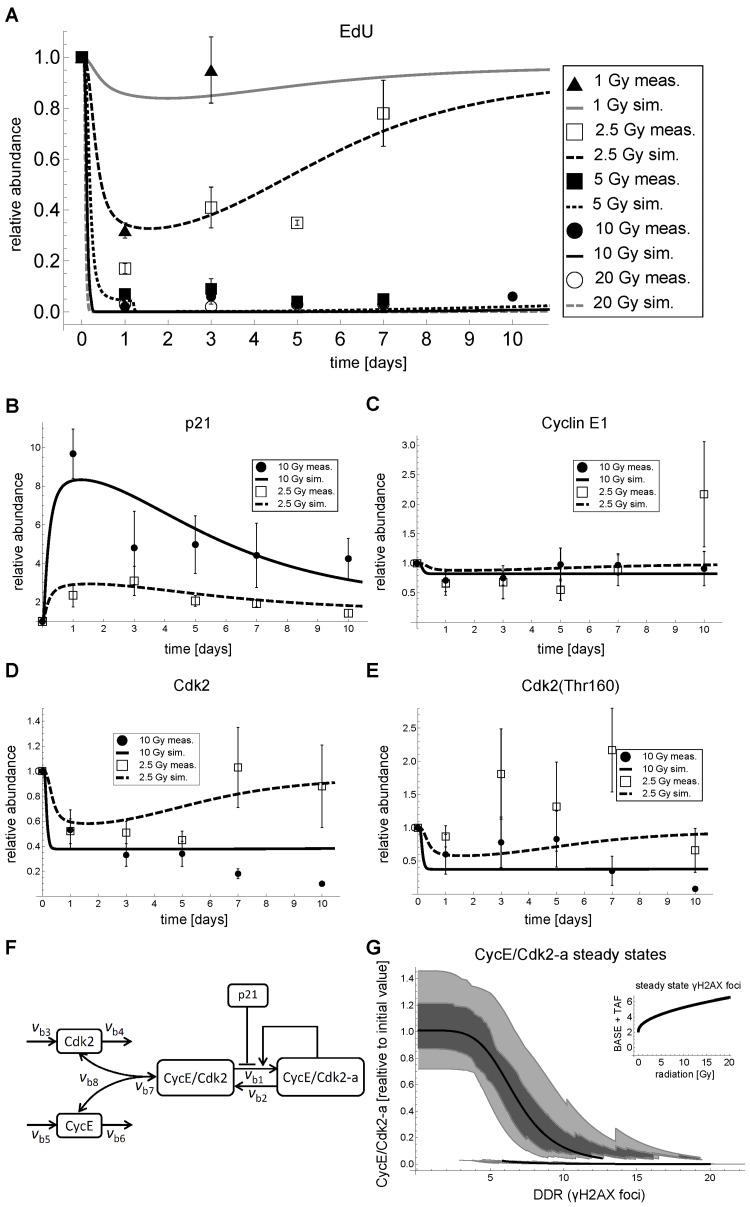
G1-S dynamics and model fits in MRC5 cells after IR. (**A**) Relative abundance of EdU positive cells and simulated active Cyclin E/Cdk2 complex (*CycECdk2-a* in panel F). (**B**) Measured and simulated relative total p21 abundance (*p21* in F).(**C**) Measured and simulated relative total Cyclin E1 abundance (*CycE*+*CycE/Cdk2*+*CycE/Cdk2-a* in panel F). (**D**) Measured and simulated relative total Cdk2 abundance (*Cdk2*+*CycE/Cdk2*+*CycE/Cdk2-a* in panel F). (**E**) Measured and simulated relative phosphorylated (Thr160) Cdk2 abundance (*CycECdk2*+*CycECdk2-a* in panel F). (**F**) Wiring scheme of the best approximating p21-dependent G1-S transition model. (**G**) Steady state analysis of active Cdk2 (*CycE/Cdk2-a* in F of the parameterized combined DNA damage-G1-S arrest model (Figure S4) as a function of DNA damage response (DDR), i.e. γH2AX foci, including free parameter perturbations by sampling 50 times from a uniform distribution within an interval of plus/minus 20% around the original parameter value. Solid line: Stable steady state of *CycE/Cdk2-a* of the parameterized model as a function of DNA damage (DDR). Light gray region: 5-95% of stable steady states of *CycE/Cdk2-a* of the parameterized model with perturbed free parameters. Dark gray region: First to third quartile of steady states of *CycE/Cdk2-a* of the parameterized model with perturbed free parameters. **Inset**: Steady state γH2AX foci, i.e. BASE+TAF from Figure S4, as a function of IR [Gy]. **A**-**D**: Lines indicate simulations of the fitted model. Symbols indicate mean measured values ± SEM (n≥3) scaled to day 0. Representative Western Blots are shown in Figure S6, Supplemental Figures. The corresponding data are provided in Supplemental Data Sets 1-13.

To identify proteins predominantly involved in the cell cycle arrest of MRC5 cells, we analyzed the activity of the p53-p21 and the p16-Rb-E2F pathway as well as the abundance of Cdc25A and important G1-S cyclins, i.e. Cyclin E1/2 and Cyclin A2, after exposure of MRC5 fibroblasts to 2.5 and 10 Gy IR (Figures 2, 3).

After 2.5 Gy and 10 Gy IR p16 seems to be transiently up-regulated. However, p16 abundance was highly variable and the patterns were not consistent (Figure 2A). This was in contrast to p21 abundance showing a consistent irradiation dose-dependent transient upregulation (Figure 3B). Moreover, the relative phosphorylation levels of the Cyclin D-Cdk4/6-specific Rb1 phosphorylation site, Ser780 [27], stayed basically unchanged (Figure 2B), indicating that Cyclin D-Cdk4/6 activity, a target of p16, is not inhibited under these conditions. Correspondingly, neither total nor the hypo-phosphorylated form of Rb1 showed a consistent pattern or substantially changed their abundance after 2.5 or 10 Gy IR (Figure 2C,D). Consequently, the Rb1-E2F regulated G1-S cyclins Cyclin E1, E2 and A2 do also not alter their abundance substantially (Figures 2E, 3C, S6). This is in line with earlier reports attributing the p16-Rb pathway mainly to replicative and oncogene-induced senescence [28]. In the following, we concentrated on Cyclin E1 as representative G1 cyclin, because Cyclin E2 was expressed at low levels and showed similar dynamics as Cyclin E1 (Figure S6).

Interestingly, also relative Cdc25A levels, which have been reported to be down-regulated after DNA damage in certain cell types [29-31], did not show a consistent down-regulation pattern (Figure 2F).

Therefore, we conclude that for 10 Gy IR and for at least the first 7 days after irradiation neither the p16-Rb1-E2F pathway nor Cdc25A down-regulation are responsible for the observed rapid and permanent G1-S arrest in MRC5 human primary fibroblasts.

#### Cdk2 is down-regulated after IR

Opposed to the commonly accepted opinion, reflected in all relevant cell cycle models we found [32-45], and as reported above, G1-S arrest after IR in MRC5 fibroblasts is not regulated at the level of cyclin abundance. Therefore, we analyzed other cell cycle related proteins and found total Cdk2 to be strongly down-regulated after 10 Gy IR, whereas for 2.5 Gy IR total Cdk2 was only transiently down-regulated (Figure 3D).

We also monitored Thr160-phosphorylated Cdk2 and found a similar, but not as clear pattern (Figure 3E). Note that the Cdk2(Thr160) antibody recognizes both active as well as inactive (additionally phosphorylated on Thr-14 and Tyr-15) Cdk2.

We hypothesized that the observed G1-S arrest after irradiation was regulated by p21-mediated Cdk2 down-regulation. We further explored this hypothesis by combining our data with mathematical models.

### Modelling DNA damage response in human primary fibroblasts after IR

#### A model for IR induced DNA damage dynamics

First, we used a simplified version of a previously described model of DNA damage response to simulate dynamics of measured γH2AX foci, a common readout for double-strand breaks [46]. For simplicity, we assumed that foci and corresponding p21 dynamics are independent from downstream processes regulating the actual G1-S arrest. Even though feedbacks between DNA damage and p21 have been reported, these feedbacks only induce short-lived DNA damage, but do not significantly contribute to long-lived (>15h) DNA damage, in which we are interested here [21]. Therefore, we developed the DNA damage-p21 module as a stand-alone model, which was used as an input for our G1-S checkpoint models (Figure 3F, Supplemental Data 2).

Existing models of DNA damage include two types of damages, i.e. fast and slowly repairable damages [47]. We extended those models by additional types of DNA damage, i.e. persistent telomere-associated foci (TAF), and by background DNA damage (BASE) (Figure S2A, Supplemental Data 2). The sum of TAF and BASE is in the following also referred to as background damage. Irradiation induced three types of DNA damages, i.e. FAST, SLOW, and TAF, which are characterized by their speed of repair, i.e. fast, slow and zero, respectively. Together with constant background DNA damage (BASE), they constitute the total amount of measured γH2AX foci, which in turn activate a signaling pathway and finally p21 (Figure S2A, Supplemental Data 2).

We parameterized this model using published data on TAF and γH2AX foci [10, 46] (Figure S2). For details on measuring and quantification of γH2AX foci time series as well as representative images refer to [46]. Combining all these data provided a data set that was suitable to parameterize our DNA damage model. In Figure S2B-F (Supplemental Data 2) time series and corresponding simulations of the parameterized DNA damage model are displayed. The parameterized model can well recapitulate time series of measured mean γH2AX foci per cell for 2.5, 10 and 20 Gy, TAF for several irradiation regimes (Figure S2, Supplemental Data 2) and p21 time series for 2.5 and 10 Gy (Figure 3B).

#### A model for IR-induced G1-S arrest dynamics after different doses of IR

Having a parameterized model for DNA damage induced p21 activation, we developed down-stream G1-S checkpoint models. Following the principle of parsimony, our models included only the most important components based on literature (Supplemental Data 1), which can explain the G1-S arrest after irradiation. We concentrated on the Cyclin E-Cdk2 complex, as this is the complex generally perceived to be responsible for regulating the G1-S transition [19, 30, 48] (Figure S1, Supplemental Data 1). Importantly, we included the possibilities of Cdk2 degradation and regulation, a feature which is absent from current cell cycle models [32-45]. We implemented and fitted several model alternatives. First, we included the possibility that Cdk2 levels were not actively regulated, but were constitutively expressed and degraded, and, second, we included the possibility that Cdk2 levels were actively regulated by some hypothetical p21-dependent mechanism. These possibilities were combined with two different mechanisms for p21-dependent inhibition of Cdk2 activity, via complex formation or activation inhibition. The wiring schemes of the six different model alternatives and their rationales are described in detail in the Supplemental Data 3 and are displayed in Figure S3. All models were fitted to the data in Figure 3, except EdU incorporation for 5 Gy, which was used for model validation. The most parsimonious model was selected using the Akaike Information Criterion (Tables S1, S2, and Supplemental Data 3). Refer to the Supplemental Data 3 for a detailed description of the model selection procedure. The most parsimonious model, which was able to recapitulate our data and had the best predictive power, is depicted in Figure 3F. Model variables are referred to in *italics* throughout the text.

The best approximating model includes constitutive synthesis and degradation of both Cdk2 (*Cdk2*) and Cyclin E (*CycE*) that is not further regulated by some hypothetical p21-dependent mechanism (reactions *v*_b3_, *v*_b5_ and *v*_b4_, *v*_b6_, respectively, Figure 3F). *Cdk2* and *CycE* can reversibly associate to build the inactive complex *(CycE/Cdk2*) (*v*_b7_, *v*_b8_). This inactive complex is then activated (*v_b_*_1_), where the active complex (*CycE/Cdk2-a*) further promotes its own activation constituting a positive feedback loop. Inactivation of the active complex is constitutive (*v*_b2_). The positive feedback in *v*_b1_ is modelled by the non-linear Goldbeter-Koshland function [49]. Such simple positive feedback motifs can show bistable behavior [50, 51] and are believed to build the core of irreversible cell cycle checkpoints [52]. Inhibition of CycE/Cdk2 activation by p21 was included as a simple reverse Hill-function. For more details on the processes underlying and motivating the model refer to Figure S1, Supplemental Data 1.

We used the DNA damage model as input for our G1-S transition model and fitted the parameters of the latter to our measured data (Figure 3). The complete model is depicted and described in detail in the Supplemental Material (Figure S4, Tables S3-S9, and Supplemental Data 4). Reasoning that the amount of active CycE/Cdk2 complex in a cell is related to the probability of a cell to enter S-phase, we used our measured EdU incorporation rates (Figure 3A) as a proxy of active CycE/Cdk2 abundance (*CycE/Cdk2-a*). Despite its simplicity, the model was able to recapitulate the main features of irradiation-induced G1-S arrest for different irradiation regimes. Moreover, the model could predict the 5 Gy EdU incorporation time series, which was used for model validation (Figure 3A). The selected model recapitulated transient and permanent down-regulation of EdU incorporation (Figure 3A), total Cdk2 (Figure 3D) and Cdk2(Thr160) (Figure 3E), after 2.5 and 10 Gy IR, respectively. The selected model has 15 free parameters and parameterized in a way that Cyclin E levels were not significantly affected by IR (Figure 3C).

Taken together, our G1-S arrest model combined with our DNA damage model (Figure S4, Supplemental Data 4) was able to explain the main features of irradiation-induced p21 mediated G1-S arrest, especially the observations that i) Cyclin E abundance is not affected by IR, ii) in contrast, Cdk2 abundance is substantially decreased after irradiation, and iii) that a IR dose of > 2.5 Gy induces a cell cycle arrest for at least 10 days.

#### The IR-induced G1-S arrest is regulated by a robust bi-stable hysteresis-switch

With the selected parameterized model we were able to quantitatively address the initial question how cells decide whether to permanently or transiently arrest in G1-S phase, i.e., to senesce or not to senesce? To this end, we analyzed steady state properties of active Cdk2 (*CycE/Cdk2-a* in Figure 3F) of our combined DNA damage-G1-S arrest model as a function of DNA damage (DDR/γH2AX foci in Figure S4, Supplemental Data 4) (Figure 3G). Figure 3G reveals that there are two branches of stable steady states (bistability) in a region from six to 12 γH2AX foci for the parameterized model (solid line in Figure 3G). Thus, the parameterized model exhibits a classical bistable hysteresis-switch. Starting from a low background DNA damage (two γH2AX foci, Figure S2F, Supplemental Data 2) Cdk2 activity (*CyE/Cdk2-a*) stays high, i.e. on the upper branch (Figure 3G, upper solid line), in case the steady-state DNA damage does not exceed 12 γH2AX foci. Even though the initial DNA damage upon 2.5 Gy IR exceeds 70 γH2AX foci (Figure S2B, Supplemental Data 2), due to time delay in the model and in conjunction with fast repair this is not sufficient to force *CycE/Cdk2-a* to switch to the lower branch (Figure 3A). Only above the threshold of 3 Gy IR *CycE/Cdk2-a* switches to the lower branch, where it stays unless the number of γH2AX foci falls to a level lower than six (Figure 3G, lower solid line). This is the case for irradiation regimes lower than 15 Gy, where the persistent γH2AX foci, i.e. base damage (BASE) and telomere-associated foci (TAF), sum up to around six γH2AX foci (Figures 3G inset, S2H, Supplemental Data 2). However, the repair process might take several days and weeks until this level is reached (Figure S2B-D, Supplemental Data 2). For irradiation regimes higher than 15 Gy γH2AX foci cannot assume levels lower than six as the TAF monotonically increase with irradiation dose (Figure 3F inset, Figure S2H, Supplemental Data 2). Consequently, the model suggests that for >15 Gy IR, Cdk2 activity stays permanently low, because of a constantly elevated DNA damage signal and, therefore, a permanent G1-S arrest is enforced.

We tested the sensitivity of the bistable behavior of our simple G1-S model by a Monte-Carlo analysis. We perturbed all free parameters including the ones from the DDR model in a range of ±20% of the original value. For each level of γH2AX foci we calculated the corresponding steady state distribution (Figure 3G, shaded regions). In principle, the bistable nature of the G1-S arrest is robust to parameter variations in the tested range. However, the bistable region varies. The lower branch of CycE/Cdk2-a steady states determines permanent G1-S arrest upon high irradiation doses, because it defines the level of DNA damage upon which Cdk2 activity can be resumed. This level varied between three and 10 γH2AX foci with a mean of six (Figure S7, Supplemental Figures). Thus, depending on the parameter set, the model can switch back to high Cdk2 activity even with elevated DNA damage of 10 γH2AX foci. Accordingly, the γH2AX foci level where high Cdk2 activity switches to low Cdk2 activity varied between seven and 20 (Figure S7, Supplemental Figures). Thus, even for high doses of irradiation there were parameter combinations, where Cdk2 activity would not switch to the lower branch and stay high even for elevated DNA damage or resume Cdk2 activity despite high background damage.

### Model predictions and dedicated follow-up experiments

#### p21 depletion is sufficient to release G1-S arrest and reconstitute Cdk2 expression

**Figure 4:**
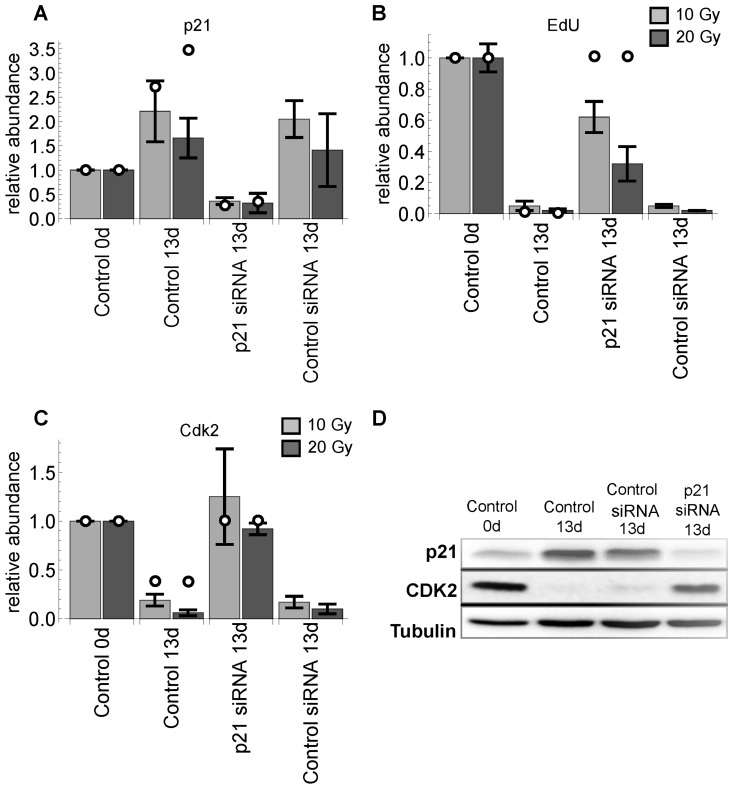
p21 silencing 11 days after IR with sample collection 2 days later (13d). (**A**) relative p21 levels. (**B**) relative EdU incorporation. (**C**) relative Cdk2 levels. All values are scaled to day 0. Light and dark gray bars indicate 10 and 20 Gy radiation, respectively. Error bars indicate standard error of the mean (SEM) (n≥3). (**D**) representative western blot demonstrating validation of the p21 silencing in irradiated MRC5 cells (20 Gy).

Blocking the DNA damage response at the level of ATM has the potential to rescue G1-S arrest [9]. It has also been shown that p53 silencing has the potential to reverse replicative senescence arrest in case of low p16 abundance [6]. Moreover, p21 is the eventual CDKI in the ATM-p53-p21 pathway (Figure S1, Supplemental Data 1). This argues for the hypothesis that p21 silencing also rescues DNA damage induced G1-S arrest. Indeed, this was predicted by our model. We corroborated these predictions by follow-up experiments, where p21 was silenced in arrested cells after 10 and 20 Gy IR. Notably, the model also predicted that after p21 depletion total Cdk2 abundance would also recover. This prediction was also verified by our follow-up experiments (Figure 4). The silencing experiment was also performed for p16 expression to verify weather this protein could have any impact on the G1-S arrest recovery. Silencing p16 did neither rescue G1-S arrest nor inhibit Cdk2 down-regulation (Figure S8, Supplementary Figures).

#### Both Cdk1 and Cdk2 down-regulation is necessary and sufficient to induce permanent G1-S arrest and senescence

**Figure 5:**
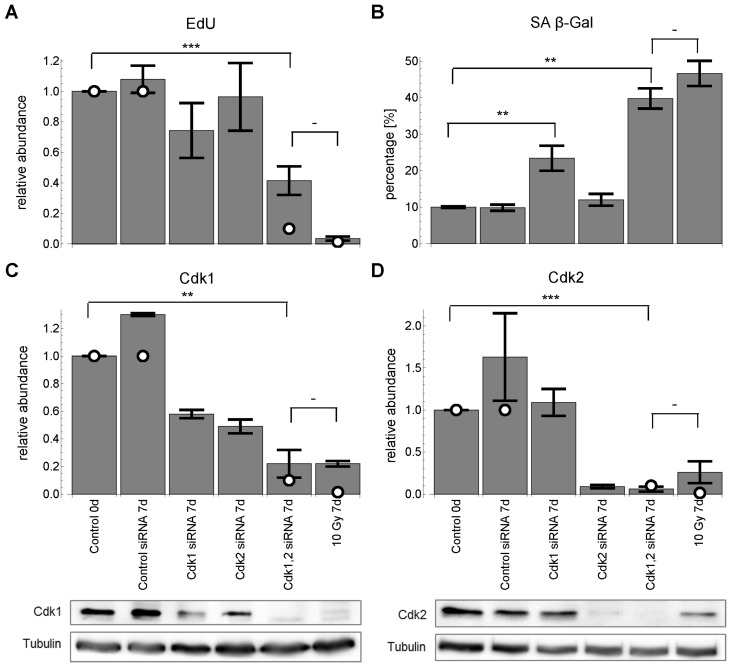
Cdk1,2 silencing for 7 days. White circles indicate corresponding model predictions. (**A**) relative EdU incorporation (mean ± SEM (n≥3)). (**B**) SA-βG activity (mean ± SEM (n≥3)). (**C**) relative Cdk1 levels (mean ± SEM (n≥3)) with representative Western blot. (**D**) relative Cdk2 levels (mean ± SEM (n≥3)) with representative Western blot. All values are scaled to control before radiation at day 0. ***: P<0.01, **: P<0.05, -: P>0.05, unpaired two-sided t-test.

Despite the fact that the model predicted Cdk2 down-regulation to be sufficient for G1-S arrest, we could not confirm this experimentally. However, the literature suggested that, at least in mouse models, Cdk1 can substitute Cdk2 at the G1-S transition [53, 54]. We could support this finding for MRC5 primary human fibroblasts showing that down-regulation of both Cdk1 and Cdk2 induced both G1/S arrest and senescence (Figure 5). Accordingly, not only Cdk2 but also Cdk1 is significantly downregulated after 10 Gy IR (Figure 5C). This finding puts into perspective the generally accepted role of Cdk2 as the only CDK controlling G1-S transition in human fibroblasts. Thus, we modified the wiring scheme of the final model indicating that both Cdk1 and Cdk2 control the G1-S transition in MRC5 fibroblasts (Figure S4, Supplemental Data 4).

#### Irradiation with 5 and 10 Gy is not sufficient to induce permanent G1-S arrest

**Figure 6:**
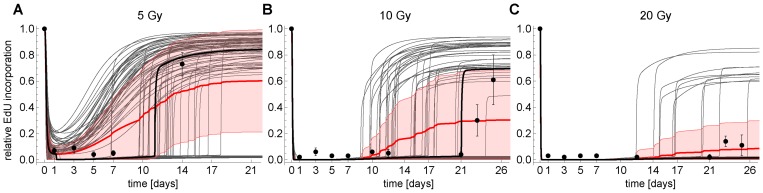
Simulated (*CycE/Cdk2-a*) and measured (EdU) time series of cells at G1-S transition for (**A**) 5 Gy IR, (**B**) 10 Gy IR and (**C**) 20 Gy IR, respectively. Thick black line: simulated *CycE/Cdk2-a* using the parameterized model (Figures 3, S4). Thin gray lines: Monte-Carlo simulation (MCS) of *CycE/Cdk2-a* using the parameterized model. Thick red line: Average MCS. Red shaded region: Standard deviation of MCS. Dots: Measured mean relative EdU incorporation ± SEM (n≥3).

The model was well able to predict swift down-regulation of EdU incorporation after 5 and 10 Gy IR. However, steady state analysis of the G1-S switch and permanent γH2AX foci indicated that for 5 and 10 Gy a transient arrest could still be possible, because the permanent DNA damage level would drop below six γH2AX foci (Figure 3G inset). Consequently, the model predicted that upon 5 and 10 Gy IR EdU incorporation would resume after 11 and 21 days, respectively (black lines in Figure 6A, B). Therefore, we conducted dedicated follow-up experiment measuring EdU incorporation after 14 and 24 days for 5 and 10 Gy IR, respectively. Indeed, EdU incorporation after 14 days upon 5Gy IR returned to around 70% of the initial rate (black dots in Figure 6A). Thus, the model correctly predicted that 5 Gy IR is not sufficient to induce permanent cell cycle arrest. In addition, 23 days after 10 Gy IR MRC5 cells exhibit a significant and sustained increase in EdU incorporation in contrast to 20 Gy irradiated cells (black dots in Figure 6B, C).

Of note, the model was parameterized using population data, and, therefore, represents a hypothetical non-existing average cell. Our Monte-Carlo analysis of the model's steady state properties indicated that even though bistability *per se* is a robust feature of the model, the range of bistability can vary (Figure 3G). Each cell in a population is different, e.g., in terms of protein concentration. We therefore addressed the question how this intercellular variability would affect DNA damage induced G1-S arrest. To this end, we mimicked G1-S arrest of single cells by another Monte-Carlo simulation, where we simulated time courses of DNA damage induced G1-S arrest varying all model parameters, including the DDR model and initial concentrations for 5, 10 and 20 Gy IR in a range of ±20% of the original value (Figure 6). For all tested conditions, there were particular parameter sets, i.e. single cells that escaped G1-S arrest. Either because they do not switch to the lower Cdk2 activity branch in the first place (for 5 Gy IR) or they switch back from the low activity to the high activity branch (Figure 6). The mean for the 10 Gy Monte-Carlo simulations showed a clear recovery of G1-S transition 14 days after IR. Thus, the model correctly predicts that even upon 10 Gy IR a substantial number of cells escape the permanent G1-S arrest. Indeed, we observed that 23 days after 10 Gy IR there was a substantial and sustained EdU incorporation (black dots in Figure 6B).

The model predicted that also after 20 Gy IR a small number of cells escape the permanent G1-S arrest, however, the mean indicated that this number is too small for a measurable signal. Indeed, 21 days after 20 Gy IR no substantial EdU incorporation was measured (black dots in Figure 6C).

#### Cell fate is determined by a combination of high initial and elevated background damage

**Figure 7:**
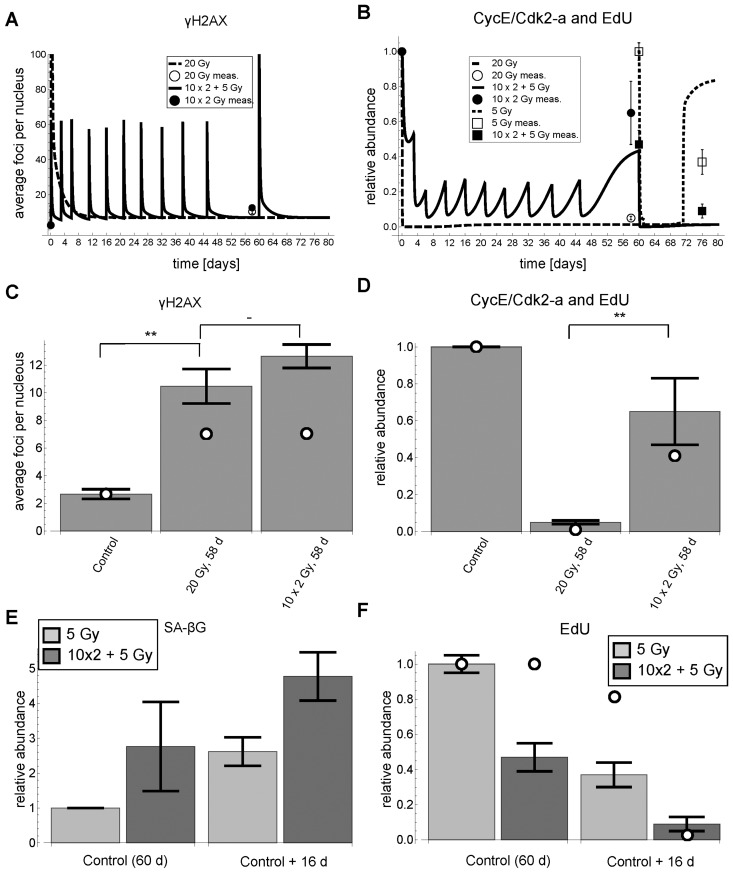
γH2AX foci, EdU incorporation and SA-βG after cumulative DNA damage. (**A**) Simulated (lines) and measured (symbols) time series of γH2AX foci (mean ± average SEM (3 rep with >50 nuclei each)), respectively. For better visibility of the cumulative damage, the y-axis was pruned at 100 foci. (**B**) Simulated (lines) and measured (symbols) time series of simulated Cdk2 activity (*CycE/Cdk2-a*) and measured EdU incorporation (mean ± SEM (n≥3)), respectively. (**C**) Simulated (open circles) and measured (bars) γH2AX foci 58 days after experiment start (Control) (mean ± average SEM (3 rep with >50 nuclei each)). White dots indicate simulated values from panel A. (**D**) Simulated Cdk2 activity (*CycE/Cdk2-a*) (open circles) and EdU incorporation (bars) (mean ± SEM (n≥3)) 58 days after experiment start (Control), respectively. White dots indicate simulated values from panel B. (**E**) SA-βG activity (mean ± SEM (n≥3)) after 5 Gy IR and 10×2+5 Gy IR 16 days after control (60 days), respectively. (**F**) EdU incorporation after 5 Gy IR and 10×2+5 Gy IR 16 days after control (60 days), respectively (mean ± SEM (n≥3)). **: P<0.05, -: P>0.1, non-paired two-sided t-test.

The model predicted that a permanently elevated DNA damage background signal keeps cells in a G1-S arrest if, and only if, the initial DNA damage was high enough to push them onto the low Cdk2 activity branch in the first place. Accordingly, the model predicted that consecutive stimuli of low irradiation would increase the permanent background damage, but fail to induce a permanent G1-S arrest and cellular senescence. To corroborate this prediction, we used the model to design an experiment of 10 consecutive irradiations of 2 Gy, accumulating to 20 Gy, where the timing of irradiations was chosen such that cells could sufficiently recover in order not to switch to the lower Cdk2 activity branch after the next irradiation. This way cells can recover Cdk2 activity after the accumulated 20 Gy, yet having the same elevated background damage as cells irradiated once with 20 Gy. Moreover, the model allowed designing such an experiment with minimal duration (Figure 7A, B).

Indeed, cells irradiated once with 20 Gy did not recover from G1-S arrest even 58 days after irradiation, whereas cells with accumulated 20 Gy did recover from G1-S arrest 58 days after the first and 13 days after the last irradiation, indicated by significantly higher EdU incorporation rates (P<0.05) (Figure 7B,D). Importantly, the background DNA damage under both irradiation regimes is not significantly different (P=0.23), i.e. around 11 γH2AX foci on average per nucleus (Figure 7A, C). These elevated background levels are probably due to mainly persistent telomeric DNA damage [9, 10]. Moreover, the measured DNA damage is well above the predicted threshold below which a cell can recover from G1-arrest after having switched to low Cdk2 activity (Figure 3G).

In addition, the model predicted that for cells harboring already elevated background damage a lower irradiation dose is sufficient for a permanent arrest. Specifically, the model predicted that in the cells with 10×2 Gy accumulated IR an additional dose of 5 Gy is sufficient to induce a permanent arrest (Figure 7A, B solid line and squared symbols). Indeed, in complete accordance with our model predictions, cells with accumulated 10×2 Gy IR arrested after additional irradiation with 5 Gy for at least 16 days with increased SA-βG activity, whereas control cells recovered 16 days after 5 Gy IR (Figures 6A, 7B, E, F).

This shows that elevated background DNA damage alone is not sufficient for permanent G1-S arrest, but that high initial DNA damage is necessary to first switch Cdk2 activity to a low state, where it can be maintained only with elevated DNA damage.

#### Cdk2-p21 ratio determines G1-S arrest

**Figure 8:**
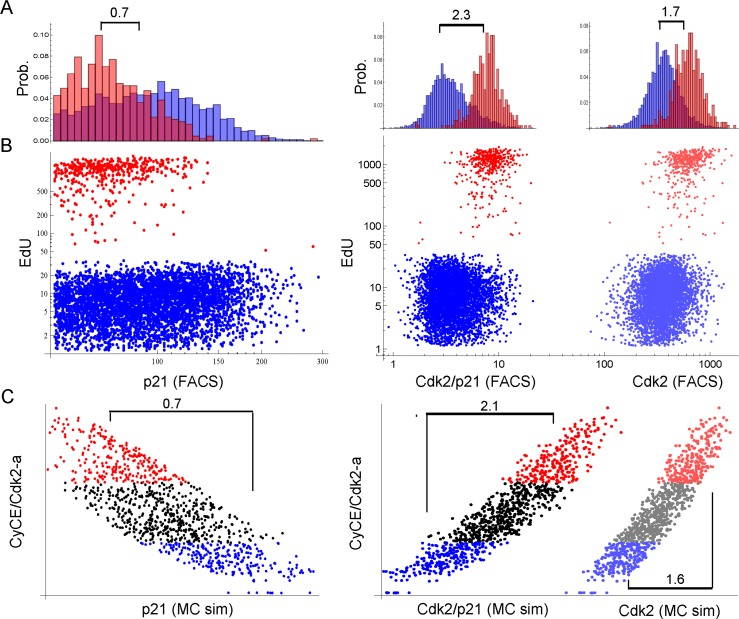
Single cell proliferation analysis of G1-S (2n) cells 3 days after 2.5 Gy IR. Cells were stained for DNA content, p21, Cdk2 and EdU. Only single 2n gated cells are shown. X-scales of A and B are equal. (**A**) Probability distribution histogram for EdU-positive (red) and EdU-negative (blue) cells. Corresponding dot plots are displayed in B. Numbers indicate the fold-difference of population means. Left panel: EdU vs. p21, Right panel: EdU vs. Cdk2/p21 and Cdk2, respectively, as indicated on the x-axis in B. (**B**) Dot plots of single cell EdU incorporation. Red: EdU-positive cells, Blue: EdU-negative cells. Left panel: EdU vs. p21, Right panel: EdU vs. Cdk2/p21 (bright dots) and Cdk2 (shaded dots), respectively. Cdk2/p21 and Cdk2 are displayed in the same graph to illustrate distribution differences. (**C**) Single cell Monte-Carlo simulation (MC sim) 3 days after 2.5 Gy IR. Red: Upper 25%-quantile of simulated active Cdk2 (CycE/Cdk2-a), Blue: Lower 25%-quantile of simulated active Cdk2 (CycE/Cdk2-a). Left panel: CycE/Cdk2-a vs. p21, Right panel: CycE/Cdk2-a vs. Cdk2/p21 (bright dots) and Cdk2 (lighter dots), respectively. Cdk2/p21 and Cdk2 are displayed in the same graph to illustrate distribution differences. Numbers indicate the fold-difference between red and blue population means.

In line with the previous observation, showing increased Cdk2 abundance upon p21 depletion (Figure 4C), the model also predicted that Cdk2 overexpression would shift the hysteresis switch in such a way that higher DNA damage or p21 levels would be necessary to switch to lower Cdk2 activities. To corroborate this prediction we designed an experiment, where we silenced p21 to different extents after 10 Gy IR and overexpressed Cdk2 concomitantly. Indeed, we observed higher EdU incorporation rates compared to control cells at similar p21 levels (Figure S9, Supplemental Figures). Thus, increased Cdk2 abundance renders p21 less effective. We further investigated how Cdk2 and p21 levels influence EdU incorporation rates. The model predicted that the Cdk2/p21 ratio is the best discriminator/predictor of EdU incorporation compared to p21 or Cdk2 levels alone. To corroborate this prediction, we measured EdU, p21 and Cdk2 levels in single G1-S (2n) cells 3 days after 2.5 Gy IR (Figure 8A,B), where cells start recovering again after transient arrest (Figure 3A).

Indeed, the factor of the population mean between EdU-positive and EdU-negative cells was largest (2.3), when Cdk2/p21 was used a predictor for EdU incorporation, compared to p21-only (0.7) or Cdk2-only (1.7) (Figure 8A). This corresponded well to the predicted factors of the Monte-Carlo simulation (Figure 8C). Thus, opposed to the currently accepted opinion, model and data support the notion that the Cdk2/p21 ratio rather than Cyclin E abundance controls G1-S arrest after DNA damage in MRC5 primary human fibroblasts.

## DISCUSSION

Cellular senescence is generally perceived as an important tumor-suppressor mechanism [4]. However, as senescent cells accumulate in tissue with age adverse effects become increasingly relevant and are believed to contribute to organismal aging [1, 55, 56]. All living beings on earth are constantly exposed to ionizing radiation (IR), which damages DNA. Usually, DNA damage is quickly repaired. However, with increasing life span the risk of obtaining persistent DNA damage in the telomeres also increases [9-11]. Therefore, one can hypothesize that persistent DNA damage induced by natural radiation contributes to the accumulation of senescent cells with age. We asked the question whether permanently elevated DNA damage is sufficient to induce senescence. The parameterized mathematical model provides a conceptually new answer to this question suggesting a mechanism how cells decide whether to permanently arrest or not. The model and corroborated predictions show that elevated background DNA damage *per se* is not sufficient to induce senescence. However, it is necessary to keep cells permanently in the arrested state. This mechanism is achieved by a bistable hysteresis switch that needs both high initial DNA damage to switch to the arrested state and elevated background DNA damage to stay there. This can be interpreted as a mechanism to prevent too many cells to prematurely senesce when they slowly accumulate persistent DNA damage. On the one hand, this might increase the risk of neoplasia with time as cells harboring substantial DNA damage keep dividing. On the other hand, we show that the stimulus needed to drive cells to senescence decreases as cells accumulate persistent DNA damage with age (Figure 7). Thus, this model provides a mechanistic explanation why the probability of obtaining senescent cells increases with age. This fits well into the view of antagonistic pleiotropy of senesce that can be beneficial early in life, but detrimental later on [4].

Moreover, the model supports the notion that both decisions either not to switch to senescence in the first place, or to switch back to proliferation despite elevated background DNA damage are subject to intrinsic molecular noise. The latter is experimentally supported by the fact that even after 10 Gy IR some cells can recover from G1-S arrest (Figure 6B). These cells most likely bear higher DNA damage than cells recovering at lower irradiation regimes. In fact, 10 Gy IR results in homogenously elevated background DNA damage indicated by low error bars in Figure S2C (Supplemental Data 2). This also demonstrates that properly parameterized models are suitable to quantitatively predict irradiation doses where the vast majority of cells would assume a certain cell fate. This can be useful for clinical applications. Our model implies that six to 12 persistent DSBs are necessary to keep cells in the arrested state. Considering only telomere-associated damage this number reduces to around 4-10. This is in line with previous reports stating that five dysfunctional telomeres are associated with replicative senescence [11].

It is a long-standing hypothesis that bistable switches control several checkpoints throughout the cell cycle [57, 58], and basically all cell cycle models employ such a mechanism [32-45]. However, we are unaware of any model for the human cell cycle that has rigorously been fitted to measured data. Our parameterized model for DNA damage regulated G1-S transition in primary human fibroblasts constitutes the first step towards this goal. We demonstrate that a model parameterized to measured data holds the key to provide conceptually new insights into human cell cycle regulation.

**Figure 9:**
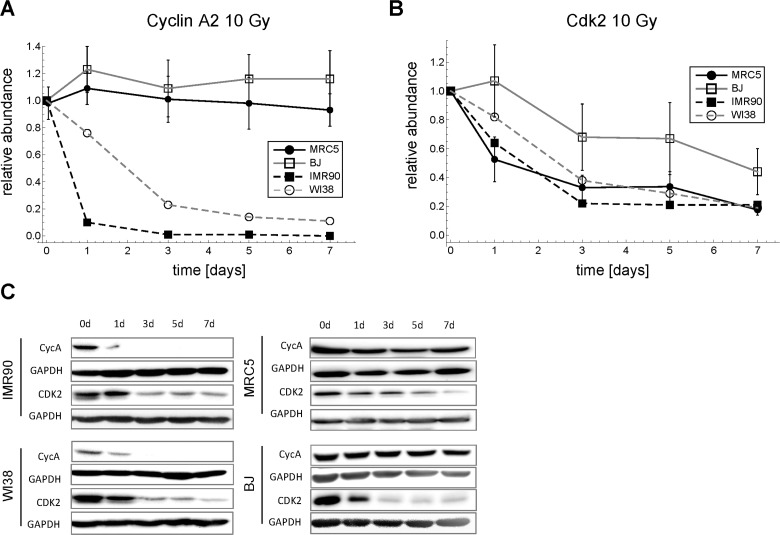
Time series of Cdk2 and Cyclin A2 expression in four different primary human fibroblasts after 10 Gy IR. (**A**) Cyclin A2 time series (mean ± SEM (n≥3)). (**B**) Cdk2 time series (mean ± SEM (n≥3)). (**C**) Representative Western blots for A and B.

Surprisingly, we found that the generally accepted view of cell cycle transitions being regulated at the level of cyclin abundance does not hold for MRC5 primary human fibroblasts after DNA damage. Basically all published cell cycle models adhere to the idea that levels of cyclin-dependent-kinases (CDKs) stay constant throughout the cell cycle [32-45] despite the fact that Cdk2 downregulation has been reported before in replicative senescence for both primary human fibroblasts [59-61] and endothelial cells [62], and Myc-induced senescence in mice [63]. Clearly, we had to abandon the concept of constant CDK levels. Surprisingly, we did not see any down-regulation of the major G1-S cyclins D, E1, E2, and A2 (Figure 2, 3, S6, Supplemental Figures), which has been reported before for WI38 and IMR90 fibroblasts [64]. Similarly to Helmbold et al. [64] we also observed down-regulation of Cyclin A2 in WI38 and IMR90 fibroblasts (Figure 9A). However, the common feature of all tested fibroblasts (MRC5, BJ, WI38, IMR90) was the substantial down-regulation of Cdk2 after irradiating cells with a dose of 10 Gy (Figure 9B). Thus, there is evidence that CDK/p21 ratio controlled DNA damage induced G1-S arrest is a general feature of primary human fibroblasts.

The model also predicted that Cdk2 overexpression alone is sufficient to release the IR-induced senescence. This prediction could only partially be corroborated (Figure S9). This argues for the involvement of additional players regulating Cdk2 activity. One of these players might be mTOR that has been shown to promote senescence, especially in the context of p53-induced senescence [65, 66]. This should be further investigated.

We also provide evidence that Cdk1 can substitute Cdk2 at the G1-S transition in MRC5 human fibroblasts (Figure 5). However, Cdk1 still seems to be the decisive kinase regulating mitosis, because down-regulating Cdk1 by half (Figure 6C) already significantly induced SA-βG activity (P<0.05) indicating senescence (Figure 6B).

Recently, it was reported that the level of Cdk2 at mitotic exit determines the proliferation-quiescence decision by a bifurcation in Cdk2 activity, which in turn is controlled by p21 [67]. Here, we show that Cdk2 abundance influences p21 effectiveness (Figure S9, Supplemental Figures), and we find a similar inverse relation between Cdk2 levels/EdU incorporation and p21 (Figure 8). Moreover, our results imply that not only the proli-feration-quiescence decision, but also the proliferation-senescence decision are controlled by Cdk2 activity. Altogether, by combining mathematical modelling with experimental data, we demonstrate that a bifurcation in Cdk2 activity regulated by the Cdk2/p21 abundance ratio controls the decision to senesce or not to senesce.

## METHODS

### Cell cultures

MRC5 primary human embryonic lung fibroblasts (ATCC, Cat. No. CCL-171™) at population doubling ~22 were cultured in Dulbecco's modified Eagle's medium (D-MEM) supplemented with 10% foetal bovine serum (FBS)(Gibco), 100 units/ml MEM non-essential amino acids solution (Gibco) and 100 units/ml penicillin, 100 μg/ml streptomycin (Gibco). Cells were grown 37°C, 95% humidity, and 5% CO_2_.

### Induction of DNA damage/cellular senescence

Cellular senescence was induced by DNA damage using γ-irradiation: human primary fibroblast cells were exposed to ionizing radiation in a Biobeam GM 2000 (Gamma Medical Service) with ^137^Cs as radioactive isotope and a dose rate of approximately 3Gy/min.

### Western blots

For Western blotting cells were harvested and lysed in RIPA-lysis buffer [50 mM Tris (pH 7.5), 150 mM NaCl, 5 mM EDTA, 10 mM K_2_HPO_4_, 10% (v/v) glycerol, 1% (v/v) Triton X-100, 0.05% SDS, 1 mM Na_3_VO_4_, 1 mM Na_2_MoO_4_, 20 mM NaF, 100μl AEBSF, 20 mM glycerol 2-phosphate and EDTA-free protease inhibitor cocktail (Roche, Mannheim, Germany)]. Cell lysates were centrifuged at 11,000 rpm, 4°C for 10 min. The concentration of total protein was determined from the supernatants using BCA protein assay kit (Pierce, Rockford, IL, USA). For western blot analysis, samples were mixed with sample buffer (250 mM Tris HCl, 5% β-mercaptoethanol, 50% glycerol, 10% SDS, 0.5% bromophenol blue), boiled for 5 min and equal amounts of protein (20 μg) were separated by sodium dodecyl sulfate polyacrylamide gel electrophoresis (12% SDS-PAGE). PVDF membranes (Roche, Mannheim, Germany) were rinsed in 100% methanol for 10 s and subsequently placed in transfer buffer (48 mM Tris, 39 mM Glycine, 0.037% SDS, 20% methanol) for 5 min. Blotting was performed at 300 mA for 2 h in a wet electroblotting system (Bio-rad). The membranes were blocked for 1 h in blocking buffer (5% skim milk) and incubated overnight at 4°C with primary antibodies. Following primary antibodies dilutions were used: Cdc2 (Cell Signaling #2546) 1:1000, Cdk2 (Thy 160) (sc-101656) 1.1000, Cdc25A (sc-56265) 1:1000, Cyc A2 (Cell Signaling #4656) 1:2000, Cyc E1 (sc-481) 1:1000, p16 (sc-468) 1:1000, p21 (Cell Signaling #2946) 1:2000, α-tubulin (Cell Signaling #3873) 1:1000. The membranes were then washed three times with PBS containing 0.1% Tween-20 and incubated with secondary antibody for 1 h. Finally, the membranes were washed three times with PBS containing 0.1% Tween-20 and the signal intensities were determined directly by using a Luminescent Imaging System (INTAS ChemoCam). The intensity of protein bands on a Western blot was quantified using *Image Studio Lite* Software 3.1 and normalized to loading control and experimental control before radiation.

### Cell count

To determine the growth rate of MRC5 cells, the cells were seeded and 24h later irradiated with the indicated dose of radiation. For cell count, the cells were harvested at indicated time points and the number of live cells was quantified with a Trypan Blue solution on an automated cell counter (Countess/Invitrogen-Life technologies).

### Annexin-V/Propidium Iodide flow cytometry analysis

Apoptosis was determined by using Annexin V-FLUOS Staining Kit (Roche) according to the manufacturer's instructions. Briefly, MRC5 primary human fibroblasts both non-radiated and irradiated either with 10 or 20 Gy were washed with 1x PBS, harvested by trypsinization and centrifuged at 200 x *g*. The cell pellet (1×10^6^) was resuspended in 100 μl of the Annexin-V-FLUOS labeling solution (2 μl Annexin-V-Fluos reagent and 2 μl Propidium iodide solution in 100 μl of incubation buffer) and incubated for 15 min at room temperature. For FACS analysis, 500 μl of the incubation buffer was added into the labeled cells. Samples were analyzed by using the CyFlow space (Partec). Positive and negative controls (incubation buffer only, Propidium iodide (PI) only, Annexin V-Fluos only) were used to set up appropriate conditions.

### Detection of SA-β-galactosidase

The SA-β-galactosidase (SA-β-Gal) assay was performed as described [68, 69] with the following modifications. To induce lysosomal alkalinization, subconfluent cells were pretreated with 300 μM chloroquine phosphate (Sigma-Aldrich) for 2 hours in fresh cell culture medium at 37°C, 5% CO_2_. Afterwards, the fluorescent substrate for SA-β-Gal (C_12_FDG, Life Technologies D-2893) was added to the cell culture medium in a final concentration of 33 μM and it was incubated for another 2 hours. At indicated experiments, during the last 45 minutes of incubation the Hoechst 33342 solution was added into the cell culture medium in a final concentration of 1 μg/ml (Life Technologies H3570). The cells were harvested by trypsinization and resuspended in PBS. Flow cytometry was performed using the CyFlow space (Partec) or the BD FACS Canto II (BD Bioscience) and data was analyzed using Flowing Software 2.5.1.

Data processing of the SA-β-galactosidase (SA-β-Gal) assay to estimate the percentage of SA-β-Gal positive cells: using negative control as a reference (non-radiated cells) the two parameter display (FSC versus C_12_FDG-FL1) was divided into two compartments by setting up a boundary between the negative (dim fluorescence) and positive cells (bright fluorescence). The percentage of positive cells was estimated by dividing the number of events within the bright fluorescence compartment by the total number of cells in the two parameter display.

### EdU incorporation assay

S-phase cells were pulse-labelled with 10 μmol/L of 5-ethynyl-2′-deoxyuridine (Click-iT EdU Alexa Fluor 488 Imaging Kit, Invitrogen) for 1h at 37°C, 5% CO_2_. EdU detection was performed according to the manufacturer's instructions. The percentage of EdU positive cells was estimated by using the two parameter display (FSC versus EdU-FL1). First, the two parameter display was divided into two compartments by setting up a boundary between the negative (dim fluorescence) and positive cells (bright fluorescence). Afterwards the percentage of positive cells was estimated by dividing the number of events within the bright fluorescence compartment for the positive cells by the total number of cells in the two parameter display.

### siRNA transfection procedure for p21 and p16

siRNA transfections were performed using SignalSilence® p21 Waf1/Cip1 siRNA I (Cell Signaling) targeting p21 protein, SignalSilence® p16 INK4A siRNA I (Cell signaling) targeting p16 protein and scrambled siRNA (Cell Signaling). MRC5 cells were transfected using RNAiMAX (Life Technologies) according to the manufacturer's protocol in a final siRNA concentration of 30 nM.

Experimental scheme of p21 and p16 silencing in irradiated cells: MRC5 cells received 10 Gy IR and then were cultured for 10 days. At 11^th^ day cells were transfected with siRNA and after two days were processed for western blot and EdU incorporation procedures described above.

### siRNA transfection procedure for Cdk1 and Cdk2

siRNA transfection was performed using SignalSilence® cdc2 siRNA I #3500 (Cell Signaling) targeting Cdk1 protein, SignalSilence® CDK2 siRNA II #7417 (Cell Signaling) targeting Cdk2 protein and scrambled siRNA (Cell signaling). MRC5 cells were transfected using RNAiMAX (Life Technologies) according to the manufacturer's protocol in a final concentration of 30 nM, except double knockdown with both Cdk1 and Cdk2, where the final concentration was 15 nM for each siRNA.

Experimental scheme of RNAi in non-irradiated cells: MRC5 cells were transfected with siRNA on the day of seeding and were reseeded at day 2 and day 5 for continuous growing. At day 7 after siRNA transfection, cells were processed for western blot.

### Flow cytometry analysis of cells labelled with EdU, DAPI, p21, and Cdk2 antibodies

S-phase cells were pulse-labelled with EdU as described above (see EdU incorporation assay). For flow cytometry cells were harvested using trypsin-EDTA, fixed with 70% ethanol. For analysis, cells were first stained with the Click-iT EdU flow cytometry assay kit, labelled with the following primary antibodies: p21 (cell signalling #2946,) and Cdk-2 (Cell Signalling #9112;) and fluorescent-labelled secondary antibodies: R-Phycoerythrin (Jackson ImmunoResearch Laboratories #115-116-068;) and Alexa Fluor 647 (cell signalling #4414;). DNA was stained with DAPI (1μg/mL, AppliChem). Flow cytometry was performed using the BD FACS Canto II (BD Bioscience) and data was analyzed using Flowing Software 2.5.1.

### Model implementation, parameterization and discrimination

Models were implemented as systems of ordinary differential equations using COPASI [70]. The free parameters were fitted to the data using COPASI's Evolutionary Programming algorithm with population size of 10 times the number of parameters, and generation number of 10 times the population size. The fitted models (Figure S3) were ranked according to the Akaike Information Criterion and the best model was selected according to its Akaike weight (Table S1, S2, Supplemental Data 3). For a detailed description of the candidate models and the selection procedure, please refer to Supplemental Data 3. The parameterized model with according data can also be found in the online Supplemental Material in COPASI- and SBML format. This model was also deposited in BioModels Database [71] and assigned the identifier MODEL1505080000.

Additional details on modelling and measurements are provided in the Supplemental Data 4 and Supplemental Methods, respectively.

## SUPPLEMENTARY MATERIAL FIGURES AND TABLES

















## References

[1] Campisi J (2005). Senescent cells, tumor suppression, and organismal aging: good citizens, bad neighbors. Cell.

[2] Collado M, Blasco MA, Serrano M (2007). Cellular senescence in cancer and aging. Cell.

[3] Kuilman T, Michaloglou C, Mooi WJ, Peeper DS (2010). The essence of senescence. Genes Dev.

[4] Campisi J (2013). Aging, cellular senescence, and cancer. Annu Rev Physiol.

[5] Lawless C, Wang C, Jurk D, Merz A, Zglinicki T, Passos JF (2010). Quantitative assessment of markers for cell senescence. Exp Gerontol.

[6] Beausejour CM, Krtolica A, Galimi F, Narita M, Lowe SW, Yaswen P, Campisi J (2003). Reversal of human cellular senescence: roles of the p53 and p16 pathways. Embo J.

[7] Stein GH, Drullinger LF, Soulard A, Dulic V (1999). Differential roles for cyclin-dependent kinase inhibitors p21 and p16 in the mechanisms of senescence and differentiation in human fibroblasts. Mol Cell Biol.

[8] Deckbar D, Jeggo PA, Lobrich M (2011). Understanding the limitations of radiation-induced cell cycle checkpoints. Crit Rev Biochem Mol Biol.

[9] Fumagalli M, Rossiello F, Clerici M, Barozzi S, Cittaro D, Kaplunov JM, Bucci G, Dobreva M, Matti V, Beausejour CM, Herbig U, Longhese MP, d'Adda di Fagagna F (2012). Telomeric DNA damage is irreparable and causes persistent DNA-damage-response activation. Nat Cell Biol.

[10] Hewitt G, Jurk D, Marques FD, Correia-Melo C, Hardy T, Gackowska A, Anderson R, Taschuk M, Mann J, Passos JF (2012). Telomeres are favoured targets of a persistent DNA damage response in ageing and stress-induced senescence. Nat Commun.

[11] Kaul Z, Cesare AJ, Huschtscha LI, Neumann AA, Reddel RR (2012). Five dysfunctional telomeres predict onset of senescence in human cells. EMBO Rep.

[12] d'Adda di Fagagna F, Reaper PM, Clay-Farrace L, Fiegler H, Carr P, Von Zglinicki T, Saretzki G, Carter NP, Jackson SP (2003). A DNA damage checkpoint response in telomere-initiated senescence. Nature.

[13] Noda A, Hirai Y, Hamasaki K, Mitani H, Nakamura N, Kodama Y (2012). Unrepairable DNA double-strand breaks that are generated by ionising radiation determine the fate of normal human cells. J Cell Sci.

[14] Verdun RE, Karlseder J (2007). Replication and protection of telomeres. Nature.

[15] Ben-Porath I, Weinberg RA (2005). The signals and pathways activating cellular senescence. Int J Biochem Cell Biol.

[16] Gil J, Peters G (2006). Regulation of the INK4b-ARF-INK4a tumour suppressor locus: all for one or one for all. Nat Rev Mol Cell Biol.

[17] Sherr CJ, McCormick F (2002). The RB and p53 pathways in cancer. Cancer Cell.

[18] Polager S, Ginsberg D (2009). p53 and E2f: partners in life and death. Nat Rev Cancer.

[19] Obaya AJ, Sedivy JM (2002). Regulation of cyclin-Cdk activity in mammalian cells. Cell Mol Life Sci.

[20] Ekholm SV, Reed SI (2000). Regulation of G(1) cyclin-dependent kinases in the mammalian cell cycle. Curr Opin Cell Biol.

[21] Passos JF, Nelson G, Wang C, Richter T, Simillion C, Proctor CJ, Miwa S, Olijslagers S, Hallinan J, Wipat A, Saretzki G, Rudolph KL, Kirkwood TB (2010). Feedback between p21 and reactive oxygen production is necessary for cell senescence. Mol Syst Biol.

[22] Bluwstein A, Kumar N, Leger K, Traenkle J, Oostrum J, Rehrauer H, Baudis M, Hottiger MO (2013). PKC signaling prevents irradiation-induced apoptosis of primary human fibroblasts. Cell Death Dis.

[23] Goldstein JC, Rodier F, Garbe JC, Stampfer MR, Campisi J (2005). Caspase-independent cytochrome c release is a sensitive measure of low-level apoptosis in cell culture models. Aging Cell.

[24] Baus F, Gire V, Fisher D, Piette J, Dulic V (2003). Permanent cell cycle exit in G2 phase after DNA damage in normal human fibroblasts. Embo J.

[25] Ye C, Zhang X, Wan J, Chang L, Hu W, Bing Z, Zhang S, Li J, He J, Wang J, Zhou G (2013). Radiation-induced cellular senescence results from a slippage of long-term G2 arrested cells into G1 phase. Cell Cycle.

[26] Johmura Y, Shimada M, Misaki T, Naiki-Ito A, Miyoshi H, Motoyama N, Ohtani N, Hara E, Nakamura M, Morita A, Takahashi S, Nakanishi M (2014). Necessary and sufficient role for a mitosis skip in senescence induction. Mol Cell.

[27] Kitagawa M, Higashi H, Jung HK, Suzuki-Takahashi I, Ikeda M, Tamai K, Kato J, Segawa K, Yoshida E, Nishimura S, Taya Y (1996). The consensus motif for phosphorylation by cyclin D1-Cdk4 is different from that for phosphorylation by cyclin A/E-Cdk2. Embo J.

[28] Serrano M (1997). The tumor suppressor protein p16INK4a. Exp Cell Res.

[29] Kastan MB, Bartek J (2004). Cell-cycle checkpoints and cancer. Nature.

[30] Busino L, Chiesa M, Draetta GF, Donzelli M (2004). Cdc25A phosphatase: combinatorial phosphorylation, ubiquitylation and proteolysis. Oncogene.

[31] Zhou BB, Elledge SJ (2000). The DNA damage response: putting checkpoints in perspective. Nature.

[32] Ling H, Kulasiri D, Samarasinghe S (2010). Robustness of G1/S checkpoint pathways in cell cycle regulation based on probability of DNA-damaged cells passing as healthy cells. Biosystems.

[33] Iwamoto K, Hamada H, Eguchi Y, Okamoto M (2011). Mathematical modeling of cell cycle regulation in response to DNA damage: exploring mechanisms of cell-fate determination. Biosystems.

[34] Iwamoto K, Tashima Y, Hamada H, Eguchi Y, Okamoto M (2008). Mathematical modeling and sensitivity analysis of G1/S phase in the cell cycle including the DNA-damage signal transduction pathway. Biosystems.

[35] Tyson JJ (1991). Modeling the cell division cycle: cdc2 and cyclin interactions. Proc Natl Acad Sci U S A.

[36] Toettcher JE, Loewer A, Ostheimer GJ, Yaffe MB, Tidor B, Lahav G (2009). Distinct mechanisms act in concert to mediate cell cycle arrest. Proc Natl Acad Sci U S A.

[37] Csikasz-Nagy A, Battogtokh D, Chen KC, Novak B, Tyson JJ (2006). Analysis of a generic model of eukaryotic cell-cycle regulation. Biophys J.

[38] Chen KC, Calzone L, Csikasz-Nagy A, Cross FR, Novak B, Tyson JJ (2004). Integrative analysis of cell cycle control in budding yeast. Mol Biol Cell.

[39] Gerard C, Tyson JJ, Novak B (2013). Minimal models for cell-cycle control based on competitive inhibition and multisite phosphorylations of Cdk substrates. Biophys J.

[40] Novak B, Kapuy O, Domingo-Sananes MR, Tyson JJ (2010). Regulated protein kinases and phosphatases in cell cycle decisions. Curr Opin Cell Biol.

[41] Novak B, Tyson JJ (2004). A model for restriction point control of the mammalian cell cycle. J Theor Biol.

[42] Gerard C, Goldbeter A (2009). Temporal self-organization of the cyclin/Cdk network driving the mammalian cell cycle. Proc Natl Acad Sci U S A.

[43] Gerard C, Goldbeter A (2014). The balance between cell cycle arrest and cell proliferation: control by the extracellular matrix and by contact inhibition. Interface Focus.

[44] Gerard C, Tyson JJ, Coudreuse D, Novak B (2015). Cell cycle control by a minimal Cdk network. PLoS Comput Biol.

[45] Ling H, Samarasinghe S, Kulasiri D (2013). Computational experiments reveal the efficacy of targeting CDK2 and CKIs for significantly lowering cellular senescence bar for potential cancer treatment. Biosystems.

[46] Rastgou Talemi S, Kollarovic G, Lapytsko A, Schaber J (2015). Development of a robust DNA damage model including persistent telomere-associated damage with application to secondary cancer risk assessment. Scientific Reports.

[47] Ma L, Wagner J, Rice JJ, Hu W, Levine AJ, Stolovitzky GA (2005). A plausible model for the digital response of p53 to DNA damage. Proc Natl Acad Sci U S A.

[48] Boutros R, Lobjois V, Ducommun B (2007). CDC25 phosphatases in cancer cells: key players?. Good targets? Nat Rev Cancer.

[49] Goldbeter A, Koshland DE (1981). An amplified sensitivity arising from covalent modification in biological systems. Proc Natl Acad Sci U S A.

[50] Tyson JJ, Chen KC, Novak B (2003). Sniffers, buzzers, toggles and blinkers: dynamics of regulatory and signaling pathways in the cell. Curr Opin Cell Biol.

[51] Ferrell JE, Xiong W (2001). Bistability in cell signaling: How to make continuous processes discontinuous, and reversible processes irreversible. Chaos.

[52] Novak B, Tyson JJ (2008). Design principles of biochemical oscillators. Nat Rev Mol Cell Biol.

[53] Aleem E, Kiyokawa H, Kaldis P (2005). Cdc2-cyclin E complexes regulate the G1/S phase transition. Nat Cell Biol.

[54] Satyanarayana A, Hilton MB, Kaldis P (2008). p21 Inhibits Cdk1 in the absence of Cdk2 to maintain the G1/S phase DNA damage checkpoint. Mol Biol Cell.

[55] Rodier F, Coppe JP, Patil CK, Hoeijmakers WA, Munoz DP, Raza SR, Freund A, Campeau E, Davalos AR, Campisi J (2009). Persistent DNA damage signalling triggers senescence-associated inflammatory cytokine secretion. Nat Cell Biol.

[56] Baker DJ, Wijshake T, Tchkonia T, LeBrasseur NK, Childs BG, van de Sluis B, Kirkland JL, van Deursen JM (2011). Clearance of p16Ink4a-positive senescent cells delays ageing-associated disorders. Nature.

[57] Novak B, Tyson JJ, Gyorffy B, Csikasz-Nagy A (2007). Irreversible cell-cycle transitions are due to systems-level feedback. Nat Cell Biol.

[58] Pomerening JR (2008). Uncovering mechanisms of bistability in biological systems. Curr Opin Biotechnol.

[59] Morisaki H, Ando A, Nagata Y, Pereira-Smith O, Smith JR, Ikeda K, Nakanishi M (1999). Complex mechanisms underlying impaired activation of Cdk4 and Cdk2 in replicative senescence: roles of p16, p21, and cyclin D1. Exp Cell Res.

[60] Lucibello FC, Sewing A, Brusselbach S, Burger C, Muller R (1993). Deregulation of cyclins D1 and E and suppression of cdk2 and cdk4 in senescent human fibroblasts. J Cell Sci.

[61] Wyllie F, Haughton M, Bartek J, Rowson J, Wynford-Thomas D (2003). Mutant p53 can delay growth arrest and loss of CDK2 activity in senescing human fibroblasts without reducing p21(WAF1) expression. Exp Cell Res.

[62] Freedman DA, Folkman J (2005). CDK2 translational down-regulation during endothelial senescence. Exp Cell Res.

[63] Campaner S, Doni M, Hydbring P, Verrecchia A, Bianchi L, Sardella D, Schleker T, Perna D, Tronnersjo S, Murga M, Fernandez-Capetillo O, Barbacid M, Larsson LG (2010). Cdk2 suppresses cellular senescence induced by the c-myc oncogene. Nat Cell Biol.

[64] Helmbold H, Komm N, Deppert W, Bohn W (2009). Rb2/p130 is the dominating pocket protein in the p53-p21 DNA damage response pathway leading to senescence. Oncogene.

[65] Leontieva OV, Blagosklonny MV (2010). DNA damaging agents and p53 do not cause senescence in quiescent cells, while consecutive re-activation of mTOR is associated with conversion to senescence. Aging (Albany NY).

[66] Korotchkina LG, Leontieva OV, Bukreeva EI, Demidenko ZN, Gudkov AV, Blagosklonny MV (2010). The choice between p53-induced senescence and quiescence is determined in part by the mTOR pathway. Aging (Albany NY).

[67] Spencer SL, Cappell SD, Tsai FC, Overton KW, Wang CL, Meyer T (2013). The proliferation-quiescence decision is controlled by a bifurcation in CDK2 activity at mitotic exit. Cell.

[68] Debacq-Chainiaux F, Erusalimsky JD, Campisi J, Toussaint O (2009). Protocols to detect senescence-associated beta-galactosidase (SA-betagal) activity, a biomarker of senescent cells in culture and in vivo. Nat Protoc.

[69] Noppe G, Dekker P, de Koning-Treurniet C, Blom J, van Heemst D, Dirks RW, Tanke HJ, Westendorp RG, Maier AB (2009). Rapid flow cytometric method for measuring senescence associated beta-galactosidase activity in human fibroblasts. Cytometry A.

[70] Hoops S, Sahle S, Gauges R, Lee C, Pahle J, Simus N, Singhal M, Xu L, Mendes P, Kummer U (2006). COPASI—a COmplex PAthway SImulator. Bioinformatics.

[71] Li C, Donizelli M, Rodriguez N, Dharuri H, Endler L, Chelliah V, Li L, He E, Henry A, Stefan MI, Snoep JL, Hucka M, Le Novere N (2010). BioModels Database: An enhanced, curated and annotated resource for published quantitative kinetic models. BMC Syst Biol.

