# Supplementary material for: To senesce or not to senesce: how primary human fibroblasts decide their cell fate after DNA damage
Source: Aging (Albany NY). 2016 Jan 30;8(1):158–76. doi: 10.18632/aging.100883 (PMC4761720; doi:10.18632/aging.100883)
Supplement: Supplementary file 1 [file aging-08-158-s001.pdf]

SUPPLEMENTAL DATA

Please browse the Full text version of this manuscript to see:
-COPASI models
-Data Sets 1-14,
-Supplemental Methods,
-Supplemental Figures
-Supplemental Tables.
