# Supplementary material for: To senesce or not to senesce: how primary human fibroblasts decide their cell fate after DNA damage
Source: Aging (Albany NY). 2016 Jan 30;8(1):158–76. doi: 10.18632/aging.100883 (PMC4761720; doi:10.18632/aging.100883)
Supplement: Supplementary file 3 [file aging-08-158-s003.docx]

### Supplemental Data 1

## DNA damage induced G1-S arrest: pathways overview


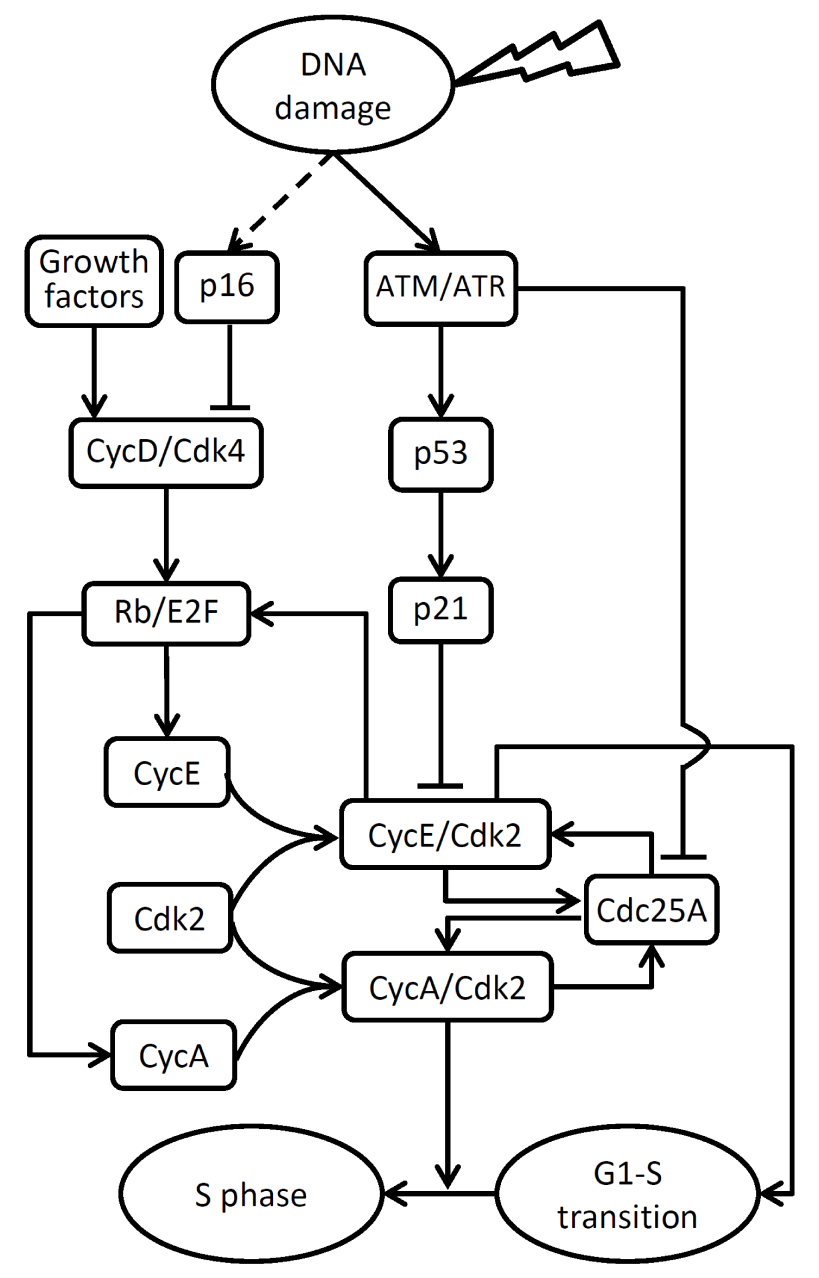


**Figure S1**: Overview of the p53-p21 pathway and the p16-Rb-E2F pathway for G1-S arrest.

At the beginning of the cell cycle growth factors lead to the formation of an active complex between cyclin D and the cyclin-dependent kinase (CDK) Cdk4/6 [[1](#_ENREF_1)]. Active Cdk4/6 phosphorylates the retinoblastoma protein Rb1, which in turn releases the Rb1-bound transcription factor family E2F1-3 [[2-4](#_ENREF_2)]. Active E2F transcription factors have been shown to trigger the transcription of important cyclins necessary for the G1-S transition and S phase, like Cyclin E and Cyclin A [[4-9](#_ENREF_4)]. Both Cyclin E and Cyclin A build a complex with the CDK Cdk2. The activated Cyclin E-Cdk2 complex is necessary to pass through the G1-S checkpoint by, e.g., further enhancing Rb1 phosphorylation and activating other E2F targets, which eventually leads to formation of active Cyclin A-Cdk2 complex and up-regulation of S phase genes and DNA synthesis [[1](#_ENREF_1)]. Like most other CDKs, the Cyclin E-Cdk2 complex requires post-translational modifications to be activated. One such modification is phosphorylation on Thr160, a highly conserved site among all CDKs, which is carried out by the CDK-activating kinase (CAK) complex [[10](#_ENREF_10)]. However, there are also counteracting modifications. Notably, the Wee1 and Myt1 kinases inhibit the kinase activity of Cdk–cyclin complexes by phosphorylating adjacent threonine and tyrosine residues in the Cdk subunit (Thr14 and Tyr15). Thus, for full Cdk2 activity these inhibitory phosphorylation have to be removed, which is accomplished by the phosphatase Cdc25A [[1](#_ENREF_1), [11-13](#_ENREF_11)]. Notably, both active Cdk2 complexes further activate their own activator Cdc25A and inhibit their own inhibitor Wee1, leading to a positive feedback [[14-18](#_ENREF_14)] and a double-negative feedback, respectively [[19](#_ENREF_19), [20](#_ENREF_20)]. These positive feedbacks involved in Cdk2 activation have been implicated in the robustness and irreversibility of the G1-S transition and S phase entry [[21-24](#_ENREF_21)]. Upon DNA damage the CDK inhibitors (CDKIs) p16 and p21 are up-regulated. The mechanism of p16 up-regulation is still unclear and is probably not directly related to DNA damage but rather a secondary effect [[25](#_ENREF_25), [26](#_ENREF_26)] (indicated by the dashed line in Figure S1). The CDKI p16 binds and inactivates the Cyclin D-Cdk4/6 complex thereby inactivating E2F transcription factors [[3](#_ENREF_3)] leading eventually to down-regulation of important G1 and S phase cyclins, especially Cyclin A [[27](#_ENREF_27)]. This way the cell cycle halts at the G1-S transition. However, the p16-Rb route to senescence seem to be important mainly in replicative and oncogene-induced senescence [[28](#_ENREF_28)]. The up-regulation of the other important CDKI p21 is directly related to DNA damage and is regulated by the transcription factor p53. DNA damage activates the DNA damage sensor ATM leading to phosphorylation and activation of p53, which in turn up-regulates p21 [[29-31](#_ENREF_29)]. Interestingly, activated ATM has also been reported to control down-regulation of the phosphatase Cdc25A through Chk1 kinase, which also de-activates Cdk2 leading to cell cycle arrest in G1 phase [[11](#_ENREF_11), [32](#_ENREF_32), [33](#_ENREF_33)]. Additionally, p21, similar to p16, binds and de-activates the Cyclin E/A-Cdk2 complexes, which is considered to be sufficient to, at least temporarily, engage the G1-S checkpoint halting the cell cycle before DNA synthesis starts [[34-36](#_ENREF_34)] (Figure S1).

**References**

1. Obaya AJ and Sedivy JM. Regulation of cyclin-Cdk activity in mammalian cells. Cell Mol Life Sci. 2002; 59(1):126-142.

2. Harbour JW, Luo RX, Dei Santi A, Postigo AA and Dean DC. Cdk phosphorylation triggers sequential intramolecular interactions that progressively block Rb functions as cells move through G1. Cell. 1999; 98(6):859-869.

3. Sherr CJ and Roberts JM. CDK inhibitors: positive and negative regulators of G1-phase progression. Genes Dev. 1999; 13(12):1501-1512.

4. Chicas A, Wang X, Zhang C, McCurrach M, Zhao Z, Mert O, Dickins RA, Narita M, Zhang M and Lowe SW. Dissecting the unique role of the retinoblastoma tumor suppressor during cellular senescence. Cancer Cell. 2010; 17(4):376-387.

5. Krek W, Ewen ME, Shirodkar S, Arany Z, Kaelin WG, Jr. and Livingston DM. Negative regulation of the growth-promoting transcription factor E2F-1 by a stably bound cyclin A-dependent protein kinase. Cell. 1994; 78(1):161-172.

6. Krek W, Xu G and Livingston DM. Cyclin A-kinase regulation of E2F-1 DNA binding function underlies suppression of an S phase checkpoint. Cell. 1995; 83(7):1149-1158.

7. Lukas C, Sorensen CS, Kramer E, Santoni-Rugiu E, Lindeneg C, Peters JM, Bartek J and Lukas J. Accumulation of cyclin B1 requires E2F and cyclin-A-dependent rearrangement of the anaphase-promoting complex. Nature. 1999; 401(6755):815-818.

8. Sherr CJ and McCormick F. The RB and p53 pathways in cancer. Cancer Cell. 2002; 2(2):103-112.

9. Timmers C, Sharma N, Opavsky R, Maiti B, Wu L, Wu J, Orringer D, Trikha P, Saavedra HI and Leone G. E2f1, E2f2, and E2f3 control E2F target expression and cellular proliferation via a p53-dependent negative feedback loop. Mol Cell Biol. 2007; 27(1):65-78.

10. Jeffrey PD, Russo AA, Polyak K, Gibbs E, Hurwitz J, Massague J and Pavletich NP. Mechanism of CDK activation revealed by the structure of a cyclinA-CDK2 complex. Nature. 1995; 376(6538):313-320.

11. Busino L, Chiesa M, Draetta GF and Donzelli M. Cdc25A phosphatase: combinatorial phosphorylation, ubiquitylation and proteolysis. Oncogene. 2004; 23(11):2050-2056.

12. Boutros R, Lobjois V and Ducommun B. CDC25 phosphatases in cancer cells: key players? Good targets? Nat Rev Cancer. 2007; 7(7):495-507.

13. Malumbres M and Barbacid M. Mammalian cyclin-dependent kinases. Trends Biochem Sci. 2005; 30(11):630-641.

14. Donzelli M and Draetta GF. Regulating mammalian checkpoints through Cdc25 inactivation. EMBO Rep. 2003; 4(7):671-677.

15. Hoffmann I, Draetta G and Karsenti E. Activation of the phosphatase activity of human cdc25A by a cdk2-cyclin E dependent phosphorylation at the G1/S transition. Embo J. 1994; 13(18):4302-4310.

16. Hoffmann I and Karsenti E. The role of cdc25 in checkpoints and feedback controls in the eukaryotic cell cycle. J Cell Sci Suppl. 1994; 18:75-79.

17. Blomberg I and Hoffmann I. Ectopic expression of Cdc25A accelerates the G(1)/S transition and leads to premature activation of cyclin E- and cyclin A-dependent kinases. Mol Cell Biol. 1999; 19(9):6183-6194.

18. Gu Y, Rosenblatt J and Morgan DO. Cell cycle regulation of CDK2 activity by phosphorylation of Thr160 and Tyr15. Embo J. 1992; 11(11):3995-4005.

19. Moroy T and Geisen C. Cyclin E. Int J Biochem Cell Biol. 2004; 36(8):1424-1439.

20. Parker LL, Sylvestre PJ, Byrnes MJ, 3rd, Liu F and Piwnica-Worms H. Identification of a 95-kDa WEE1-like tyrosine kinase in HeLa cells. Proc Natl Acad Sci U S A. 1995; 92(21):9638-9642.

21. Yao G, Lee TJ, Mori S, Nevins JR and You L. A bistable Rb-E2F switch underlies the restriction point. Nat Cell Biol. 2008; 10(4):476-482.

22. Yao G, Tan C, West M, Nevins JR and You L. Origin of bistability underlying mammalian cell cycle entry. Mol Syst Biol. 2011; 7:485.

23. Skotheim JM, Di Talia S, Siggia ED and Cross FR. Positive feedback of G1 cyclins ensures coherent cell cycle entry. Nature. 2008; 454(7202):291-296.

24. Tyson JJ. Modeling the cell division cycle: cdc2 and cyclin interactions. Proc Natl Acad Sci U S A. 1991; 88(16):7328-7332.

25. Herbig U, Jobling WA, Chen BP, Chen DJ and Sedivy JM. Telomere shortening triggers senescence of human cells through a pathway involving ATM, p53, and p21(CIP1), but not p16(INK4a). Mol Cell. 2004; 14(4):501-513.

26. Simboeck E, Gutierrez A, Cozzuto L, Beringer M, Caizzi L, Keyes WM and Di Croce L. DPY30 regulates pathways in cellular senescence through ID protein expression. Embo J. 2013; 32(16):2217-2230.

27. Helmbold H, Komm N, Deppert W and Bohn W. Rb2/p130 is the dominating pocket protein in the p53-p21 DNA damage response pathway leading to senescence. Oncogene. 2009; 28(39):3456-3467.

28. Serrano M. The tumor suppressor protein p16INK4a. Exp Cell Res. 1997; 237(1):7-13.

29. Bakkenist CJ and Kastan MB. DNA damage activates ATM through intermolecular autophosphorylation and dimer dissociation. Nature. 2003; 421(6922):499-506.

30. Shiloh Y. ATM and related protein kinases: safeguarding genome integrity. Nat Rev Cancer. 2003; 3(3):155-168.

31. Shimada M and Nakanishi M. DNA damage checkpoints and cancer. J Mol Histol. 2006; 37(5-7):253-260.

32. Kastan MB and Bartek J. Cell-cycle checkpoints and cancer. Nature. 2004; 432(7015):316-323.

33. Zhou BB and Elledge SJ. The DNA damage response: putting checkpoints in perspective. Nature. 2000; 408(6811):433-439.

34. Beausejour CM, Krtolica A, Galimi F, Narita M, Lowe SW, Yaswen P and Campisi J. Reversal of human cellular senescence: roles of the p53 and p16 pathways. Embo J. 2003; 22(16):4212-4222.

35. McConnell BB, Starborg M, Brookes S and Peters G. Inhibitors of cyclin-dependent kinases induce features of replicative senescence in early passage human diploid fibroblasts. Curr Biol. 1998; 8(6):351-354.

36. Campisi J. Aging, cellular senescence, and cancer. Annu Rev Physiol. 2013; 75:685-705.
