# Supplementary material for: To senesce or not to senesce: how primary human fibroblasts decide their cell fate after DNA damage
Source: Aging (Albany NY). 2016 Jan 30;8(1):158–76. doi: 10.18632/aging.100883 (PMC4761720; doi:10.18632/aging.100883)
Supplement: Supplementary file 4 [file aging-08-158-s004.docx]

### Supplemental Data 2

## DNA damage response model

**
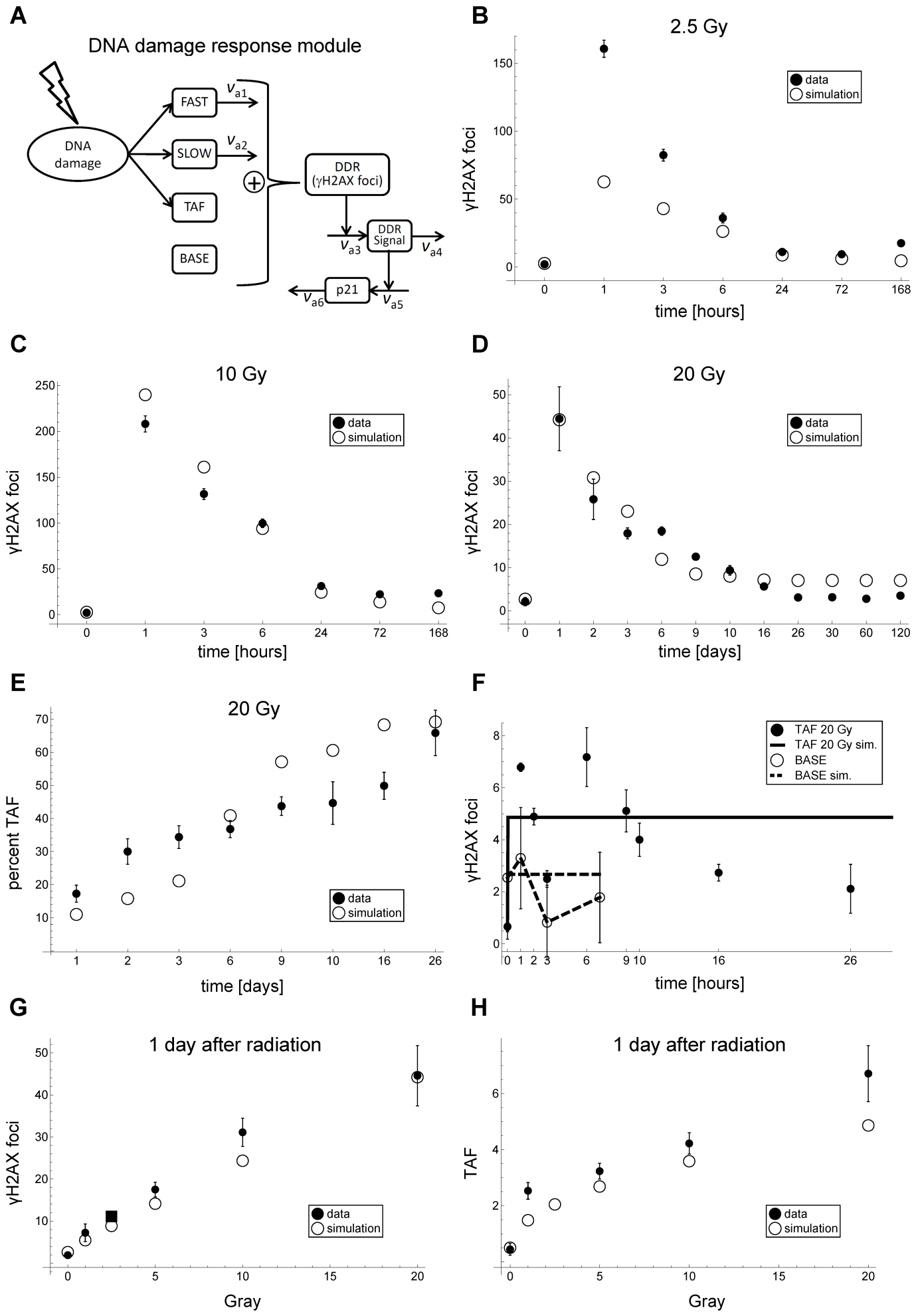
**

**Figure S2**: The DNA damage response module. **A**) Wiring scheme of the DNA damage response (DDR) model including DDR signalling and p21. **B**) Measured ± SE (n>50) (dots) and simulated (open circles) mean number of γH2AX foci per cell for 2.5 Gy IR. For a better overview the time points are not equidistant. **C**) Measured ± SE (n>50) (dots) and simulated (open circles) mean number of γH2AX foci per cell for 10 Gy IR. For a better overview the time points are not equidistant. **D**) Measured ± SE (n>50) (dots) and simulated (open circles) mean number of γH2AX foci per cell for 20 Gy IR. For a better overview the time points are not equidistant. **E**) Measured ± SEM (dots) and simulated (solid lines) mean percent telomere associated foci (TAF) per cell for 20 Gy IR. **F**) Measured (dots) and simulated (solid line) TAF and measured (open circles) and simulated (dashed line) γH2AX foci for 0Gy IR (BASE). **G**) Measured ± SEM (dots) and simulated (open circles) mean number of γH2AX foci per cell after one day for different irradiation. The closed square indicates estimated γH2AX foci from a linear interpolation used to scale our measured data (see Supplementary Methods Section) **H**) Measured ± SEM (dots) and simulated (open circles) mean number of TAF per cell after one day for different irradiation. Data in panels **D**-**H** are digitized from Hewitt et al. (2012). Data in panels **B** and **C** are taken from Rastgou Talemi et al. (2014). Details on data quantification and normalization as well as representative images can also be found in Rastgou Talemi et al. (2014). SE indicates standard error (n≥50) and SEM indicates standard error of the mean (n≥50).
