# Supplementary material for: To senesce or not to senesce: how primary human fibroblasts decide their cell fate after DNA damage
Source: Aging (Albany NY). 2016 Jan 30;8(1):158–76. doi: 10.18632/aging.100883 (PMC4761720; doi:10.18632/aging.100883)
Supplement: Supplementary file 5 [file aging-08-158-s005.docx]

### Supplemental Data 3

## DNA damage induced G1-S arrest: candidate models and selection

We developed a set of simple mathematical G1-S transition models, which could potentially explain our data. Specifically, we wanted to test a) whether the level of total Cdk2 was actively down-regulated or not, and b) which mechanism of p21-inhibition could explain our data.

The following reactions constitute the core G1-S module, which was the same for all model candidates (Figure S3): 1) reversible activation of the CycE/Cdk2-complex (reactions *v*_b1_ and *v*_b2_), where activation (*v*_b1_) is modified by an active Cdk2 (CycE/Cdk2-a) mediated positive feedback using a Goldbeter-Koshland function [[1](#_ENREF_1), [2](#_ENREF_2)]; 2) reversible binding of Cdk2 and CycE to the inactive CycE/Cdk2-complex (reactions *v*_b7_ and *v*_b8_) ; 3) constitutive production and degradation of Cdk2, respectively (reactions *v*_b3_ and *v*_b4_).

The candidate models differed the way in which Cdk2 activation was inhibited, and the way Cdk2 levels were regulated. We employ two different ways in which Cdk2 activation was inhibited, first, by a generic mechanism, where the activation reaction (*v*_b1_) was inhibited by p21 using a reverse Hill-kinetic (Figure S3, left panel, Models 1, 3, 5), and, second, by reversible binding of four p21 molecules to the non-active CycE/Cdk2-complex (reactions *v*_b5_ and *v*_b6_) (Figure S3, right panel, Models 2, 4, 6). Models with the second alternative omitted constitutive CycE production and degradation, because otherwise no switch-like steady-state behaviour can be achieved. Each of the above described options had three different ways in which Cdk2 level was regulated: i) constitutive production and degradation only (Models 1, 2), ii) p21-mediated degradation with mass action kinetics (Models 3, 4), and, iii) p21-inhibited production with reverse Hill-kinetics (Models 5, 6). The combination of all these alternatives resulted in six different models, whose wiring schemes (without the DNA damage module) are depicted in Figure S3.


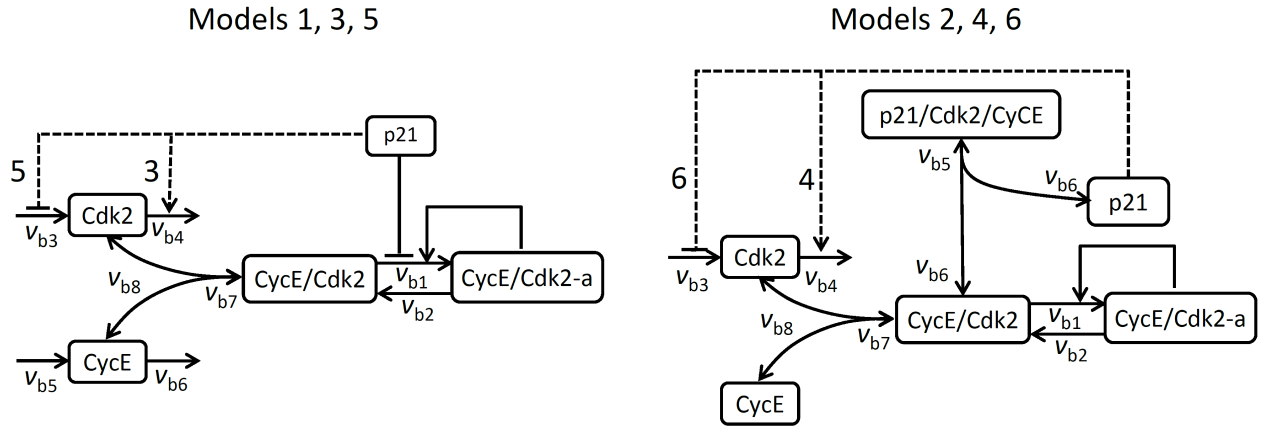


**Figure S3**: Wiring schemes of the candidate models for the G1-S arrest model without the DNA-damage module, which was the same for all candidates. Dashed lines indicate alternative regulation mechanisms and respective model numbers.

For model ranking, we calculated the Akaike Information Criterion corrected for small sample sizes (*AICc*) [[3](#_ENREF_3)] for each candidate model:

,

where *SSR* is the sum of squared residuals of the fit, *k* the number of parameters, and *n* the number of data points. The *AICc* is an information-theory based measure of parsimonious data representation that incorporates the goodness of the fit (*SSR*) as well as the complexity of the model (*k*), thereby giving an objective measure for model selection and discrimination. Using the *AICc* the models were ranked according their data approximation (Table S1, S2).

In order to select and compare the best approximating model(s) we calculated the Akaike weights (*AICw*) [[3](#_ENREF_3)].

${AICw}_{i}=\frac{e^{-\frac{1}{2}\Delta_{i}}}{\sum_{r=1}^{R} e^{-\frac{1}{2}\Delta_{r}}}$ ,

where $\Delta_{i}={AIC}_{i}-{AIC}_{min}$, with *i* being the model index *i*=1, …, 6 according to ranking and ${AIC}_{min}$ the minimal *AICc*. The *AICw’s* can be considered as the weight of evidence in favour of a model given as a number between 0 and 1, i.e. the higher the weight, the closer the model is to the hypothetical true model. We considered those models as best approximating that had an *AICw > 0.125* (Cutoff) [[3](#_ENREF_3)]*.* Taking into account the model fits Model 1 is ranked first, however, all models with Hill-inhibition fit the data similarly well and are within the cutoff (Table S1). To come to better discrimination between the models we included the prediction of the 5Gy IR EdU time series (Figure 4A) into the SSR without refitting and re-ranked the models (Table S2). Clearly, Model Nr. 1 was now selected as the model having both a good fit to the data and superior prediction properties.

**Table S1**: Candidate models according to AICc.

| **Rank** | **Model Nr.** | ***n*** | ***k*** | ***SSR*** | ***AICc*** | **Akaike weight** | **Cutoff** |
| --- | --- | --- | --- | --- | --- | --- | --- |
| 1 | 1 | 75 | 15 | 8.74 | 85.30 | 0.547 | OK |
| 2 | 3 | 75 | 17 | 8.95 | 87.03 | 0.23 | OK |
| 3 | 5 | 75 | 15 | 8.34 | 87.23 | 0.208 | OK |
| 4 | 6 | 75 | 16 | 9.14 | 92.82 | 0.013 | - |
| 5 | 4 | 75 | 14 | 10.21 | 95.83 | 0.003 | - |
| 6 | 2 | 75 | 14 | 16.35 | 134.47 | 0 | - |

*AICc*: corrected Akaike information criterion, *SSR*: sum of squared residuals, *n* number of data points, *k* number of fitted parameters.

**Table S2**: Candidate models according to AICc including 5Gy EdU prediction.

| **Rank** | **Model Nr.** | ***n*** | ***k*** | ***SSR*** | ***AICc*** | **Akaike weight** | **Cutoff** |
| --- | --- | --- | --- | --- | --- | --- | --- |
| 1 | 1 | 82 | 15 | 8.84 | 86.04 | 0.993 | OK |
| 2 | 6 | 82 | 17 | 9.50 | 95.93 | 0.007 | - |
| 3 | 4 | 82 | 15 | 11.55 | 106.02 | 0 | - |
| 4 | 3 | 82 | 16 | 11.44 | 107.20 | 0 | - |
| 5 | 5 | 82 | 14 | 10.97 | 109.76 | 0 | - |
| 6 | 2 | 82 | 14 | 19.59 | 149.31 | 0 | - |

*AICc*: corrected Akaike information criterion, *SSR*: sum of squared residuals, *n* number of data points, *k* number of fitted parameters.

Thus, Model Nr. 1, i.e. without p21-mediated Cdk2 regulation and Cdk2 activation inhibition, was selected and used for further analyses. The complete selected model is again depicted Figure S4 and described in details in the following section.

**References**

1. Goldbeter A and Koshland DE, Jr. An amplified sensitivity arising from covalent modification in biological systems. Proc Natl Acad Sci U S A. 1981; 78(11):6840-6844.

2. Tyson JJ, Chen KC and Novak B. Sniffers, buzzers, toggles and blinkers: dynamics of regulatory and signaling pathways in the cell. Curr Opin Cell Biol. 2003; 15(2):221-231.

3. Burnham KP and Anderson DR. (2002). Model Selection and Multi-Model Inference: A Practical Information-theoretic Approach. (New York: Springer).
