# Supplementary material for: To senesce or not to senesce: how primary human fibroblasts decide their cell fate after DNA damage
Source: Aging (Albany NY). 2016 Jan 30;8(1):158–76. doi: 10.18632/aging.100883 (PMC4761720; doi:10.18632/aging.100883)
Supplement: Supplementary file 6 [file aging-08-158-s006.docx]

### Supplemental Data 4

## DNA damage induced G1-S arrest: the selected model

Here, we describe the final selected combined model including the DNA damage response (DDR) model (Figure S2A) and the G1-S arrest model (Figure 4F) that was used for G1-S arrest simulations and predictions (Figures 4-9). The wiring scheme of the selected model is depicted here for convenience.


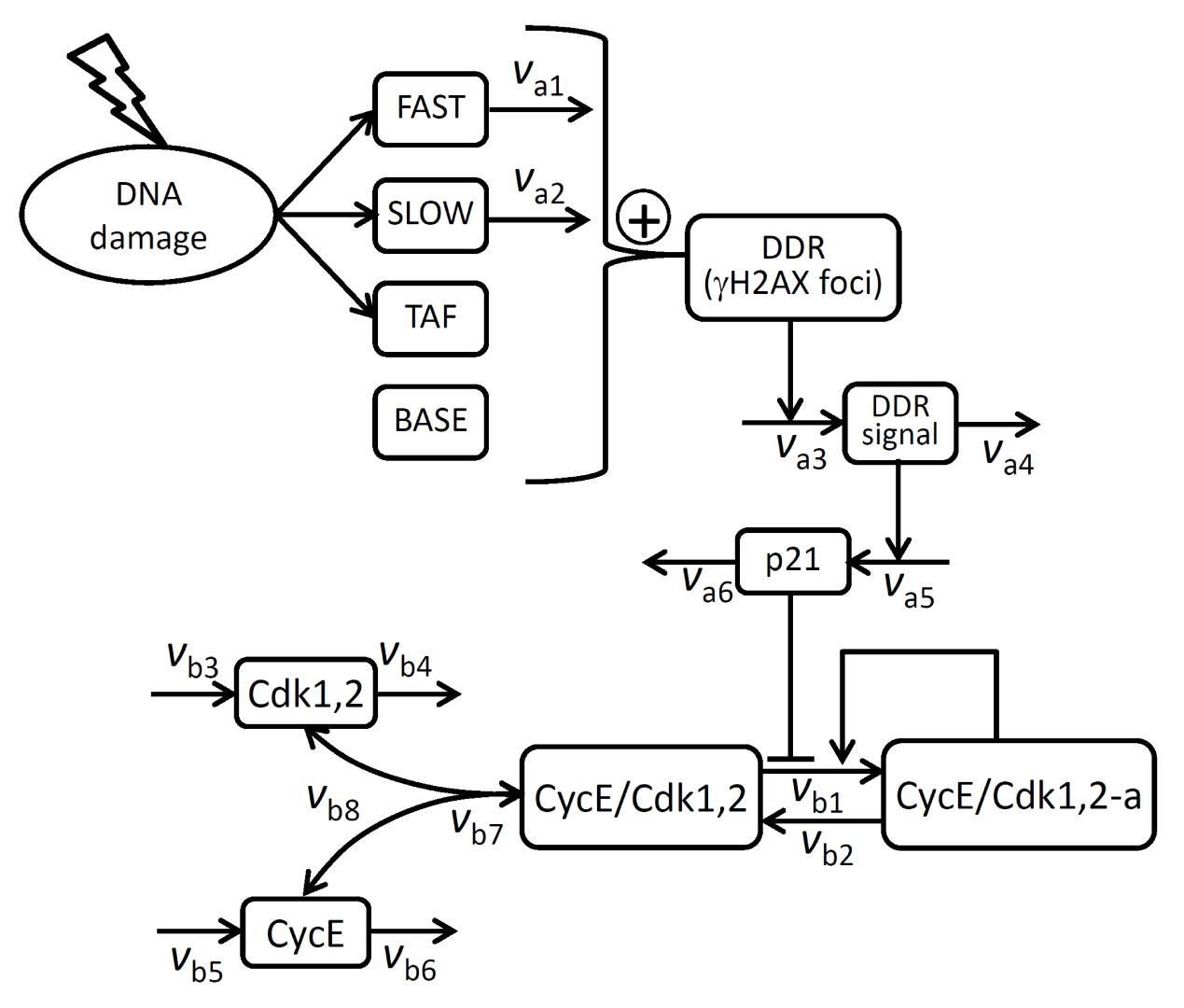


**Figure S4**: The wiring scheme of the best approximating combined DDR-G1-S arrest model.

The model (Supplementary Tables S3-S9) can be downloaded as a COPASI-file (Version 4.7, Build 34) from the Online Supplementary Material, zipped together with the corresponding data for fitting and prediction (Figure 4 in the main text). This model was also deposited in BioModels Database [[1](#_ENREF_1)] and assigned the identifier MODEL1505080000. COPASI is freely available from www.copasi.org. Extract the model and the data files into the same directory such that model fits and predictions can be reproduced by running the parameter estimation task with the method ‘Current Solution Statistics’.

**Table S3**: State variables and their initial conditions. **Bold** values are estimated parameters ± asymptotic standard deviation. All values have been rounded.

| **Model** | **Component** | **Initial Condition** | **Remark** |
| --- | --- | --- | --- |
| DDR Model | *FAST*  [#] | 0 | DNA damage that is quickly repaired. Induced by IR, adds to the measured number of γH2AX foci. |
|  | *SLOW* [#] | 0 | DNA damage that is slowly repaired. Induced by IR, adds to the measured number of γH2AX foci. |
|  | *TAF*  [#] | **0.5±0.88** | Telomere associated DNA damage foci. Induced by IR, adds to the measured number of γH2AX foci. |
|  | *p21*  [-] | 1 | p21 concentration, relative to base level. |
|  | *DDRSignal* [-] | 1 | DNA damage signal relative to base level. |
|  | *DDR*  [#] | 2.67 | DNA damage response, measured by number of γH2AX foci. |
| G1-S Model | *Cdk2* [a.u.] | **0.017±3.72** | Free Cdk2. |
|  | *CycE*  [a.u.] | **9.22±2036** | Free Cyclin E. |
|  | *CycECdk2*  [a.u.] | **1.37±301** | Inactive Cyclin E/Cdk2 complex. |
|  | *CycECdk2-a*  [a.u.] | **2.28±503** | Active Cyclin E/Cdk2 complex. |

**Table S4**: Events. [*IR*] denotes the absorbed ionizing radiation [Gy], i.e. the DNA damage inducing stimulus triggering *p21* production and subsequent G1-S arrest.indicates initial condition.

| **Component** | **Trigger Event** | **Trigger function** |
| --- | --- | --- |
| *FAST*  [#] | t>0 | $(1-f_{SLOW})[IR]p_{FociperGy}$ |
| *SLOW* [#] | t>0 | $f_{SLOW}[IR]p_{FociperGy}$ |
| *TAF*  [#] | t>0 | $[TAF]_{0}+k_{TAF}\sqrt{[IR]}$ |

**Table S5**: Ordinary algebraic-differential equation system. Concentrations are denoted by [].

| **Model** | **ODEs** |
| --- | --- |
| DDR Model | $\frac{d[FAST]}{dt}=-v_{a1}$ |
|  | $\frac{d[SLOW]}{dt}=-v_{a2}$ |
|  | $\frac{d[TAF]}{dt}=0$ |
|  | $[DDR]=p_{BaseDamage}+[FAST]+[SLOW]+[TAF]$ |
|  | $\frac{d[DDRSignal ]}{dt}=v_{a3}-v_{a4}$ |
|  | $\frac{d[p21]}{dt}=v_{a5}-v_{a6}$ |
| G1-S Model | $\frac{d[CycECdk2\text{-a}]}{dt}=v_{b1}-v_{b2}$ |
|  | $\frac{d[CycECdk2]}{dt}=-v_{b1}+v_{b2}+v_{b7}-v_{b8}$ |
|  | $\frac{d[Cdk2]}{dt}=v_{b3}-v_{b4}-v_{b7}+v_{b8}$ |
|  | $\frac{d[CycE]}{dt}=v_{b5}-v_{b6}-v_{b7}+v_{b8}$ |

**Table S6**: Rate equations of the model. Concentrations are denoted by []. Estimated parameters and derived parameters are described in Tables S6 and S7, respectively.

| **Model** | **Rate** | **Rate equation** | **Description and Rationale** |
| --- | --- | --- | --- |
| DDR Model | $v_{a1}$ | $k_{1a}[FAST]$ | DNA damage is repaired following a mass action kinetics. Note that upon DNA damage $[FAST]$is set by an event. |
|  | $v_{a2}$ | $k_{2a}[SLOW]$ | DNA damage is repaired following a mass action kinetics. Note that upon DNA damage $[SLOW]$is set by an event. |
|  | $v_{a3}$ | $k_{3a}[DDR]$ | *DDRSignal* is activated depending on the amount of DNA damage. |
|  | $v_{a4}$ | $k_{4a}[DDRSignal ]$ | *DDRSignal* is constitutively de-activated. |
|  | $v_{a5}$ | $k_{5a}[DDRSignal ]$ | *DDRSignal* activates p21. |
|  | $v_{a6}$ | $k_{6a}[p21]$ | p21 is constitutively de-activated. |
| G1-S Model | $v_{b1}$ | $\frac{[CycECdk2]\left(k_{b0}+k_{b1}G(k_{b1x}[CycECdk2\text{-}a],k_{b1y},K_{mx},K_{my}\right)}{1+\left(K_{bi}\left[p21\right]\right)^{h_{bi}}}$ | CycECdk2-a is a) constitutively activated by *k*_0_, b) activated by positive auto-regulation involving the Goldbeter-Koshland function, mimicking the positive feedback involving Cdc25A [[2](#_ENREF_2)], and c) inhibited by p21. |
|  | $v_{b2}$ | $k_{b2}[CycECdk2\text{-}a]$ | CycECdk2-a is constitutively deactivated |
|  | $v_{b3}$ | $k_{b3}$ | Cdk2 is constitutively produced |
|  | $v_{b4}$ | $k_{b4}[Cdk2]$ | Cdk2 is constitutively degraded |
|  | $v_{b5}$ | $k_{b5}$ | CycE is constitutively produced |
|  | $v_{b6}$ | $k_{b6}[CycE]$ | CycE is constitutively degraded |
|  | $v_{b7}$ | $k_{b7}[CycE][Cdk2]$ | CycECdk2 complex association |
|  | $v_{b8}$ | $k_{b8}[CycECdk2]$ | CycECdk2 complex dissociation |

$G(u,v,J,K)=\frac{2uK}{v-u+vJ+uK+\sqrt{\left(v-u+vJ+uK\right)^{2}-4uK(v-u)}}$ is the Goldbeter-Koshland function [[2](#_ENREF_2), [3](#_ENREF_3)].

**Table S7**: Estimated parameter values ± asymptotic standard deviation. Parameters for the DDR model (Figure S2A) and for the G1-S arrest model (Figure 4F in the main text) were separately estimated. Estimated initial conditions are listed in Table S3.

| **Model** | **Parameter** | **Value** | **Description** |
| --- | --- | --- | --- |
| DDR model | $p_{BaseDamage}$ | 2.16±1.00 | Background DNA damage independent from radiation |
|  | $p_{FociperGy}$ | 28.97±2.26 | Total induced γH2AX foci per Gy IR. |
|  | $f_{SLOW}$ | 0.09±0.035 | Fraction of slowly repairable γH2AX |
|  | $k_{TAF}$ | 0.97±0.28 | Parameter determining how many TAFs per Gy IR are induced |
|  | $k_{a1}$ | 0.235±0.04 | $v_{a1}$ reaction rate constant |
|  | $k_{a2}$ | 0.0165±0.007 | $v_{a2}$ reaction rate constant |
|  | $k_{a3}$ | 0.0055±0.003 | $v_{a3}$ reaction rate constant |
|  | $k_{a5}$ | 193.2±605301 | $v_{a5}$ reaction rate constant |
| G1-S arrest model | $k_{b0}$ | 0.10249 ± 14.08 | $v_{b1}$ reaction rate constant, constitutive CycECdk2-activation |
|  | $k_{b1}$ | 4.0 ± 514 | $v_{b1}$ reaction rate constant |
|  | $k_{b1x}$ | 3.4 ± 7216 | $v_{b1}$ reaction rate constant |
|  | $K_{mx}$ | 0.001 ± 3.7 | $v_{b1}$ reaction rate constant |
|  | $k_{b1y}$ | 0.325 ± 87 | $v_{b1}$ reaction rate constant |
|  | $K_{my}$ | 0.008 ± 26 | $v_{b1}$ reaction rate constant |
|  | $K_{bi}$ | 0.39 ± 0.03 | $v_{b1}$ p21-dependent inhibition constant |
|  | $h_{bi}$ | 4.9 ± 2.4 | $v_{b1}$Hill-constant for p21-dependent inhibition |
|  | $k_{b3}$ | 99.84± 193421 | $v_{b3}$ reaction rate constant |
|  | $k_{b5}$ | 10 ± 2528 | $v_{b5}$ reaction rate constant |
|  | $k_{b7}$ | 10 ± 2123 | $v_{b7}$ reaction rate constant |

**Table S8**: Derived Parameters. Assuming an initial steady state several parameters can be derived. indicates initial condition.

| **Model** | **Parameter** | **Definition** | **Description** |
| --- | --- | --- | --- |
| DDR model | $k_{a4}$ | $\left[v_{a3}\right]_{0}/[p53]_{0}$ | $v_{a3}$ reaction rate constant |
|  | $k_{a6}$ | $\left[v_{a5}\right]_{0}/[p21]_{0}$ | $v_{a5}$ reaction rate constant |
| G1-S arrest model | $k_{b2}$ | $\frac{\left[v_{b1}\right]_{0}}{[CycECdk2\text{-}a]_{0}}$ | $v_{b2}$ reaction rate constant |
|  | $k_{b4}$ | $\frac{\left[v_{b3}\right]_{0}}{[Cdk2]_{0}}$ | $v_{b4}$ reaction rate constant |
|  | $k_{b6}$ | $\frac{\left[v_{b5}\right]_{0}}{[CycE]_{0}}$ | $v_{b6}$ reaction rate constant |
|  | $k_{b8}$ | $\frac{\left[v_{b7}\right]_{0}}{[CycECdk2]_{0}}$ | $v_{b8}$ reaction rate constant |

**Table S9**: Auxiliary variables for data fitting.

| **Variable** | **Definition** | **Description** |
| --- | --- | --- |
| *Fit_Cdk2_T* | $\frac{[Cdk2]+[CycECdk2]+[CycECdk2\text{-}a]}{[Cdk2]_{0}+[CycECdk2]_{0}+[CycECdk2\text{-}a]_{0}}$ | Relative total Cdk2. Used to fit Cdk2. |
| *Fit_Cdk2_Thr160* | $\frac{[CycECdk2]+[CycECdk2\text{-}a]}{[CycECdk2]_{0}+[CycECdk2\text{-}a]_{0}}$ | Relative Thr160-phosphorylated Cdk2. Comprises all Complexed Cdk2. Used to fit Thr160-phosphorylated Cdk2. |
| *Fit_Cdk2-a* | $\frac{[CycECdk2\text{-}a]}{[CycECdk2\text{-}a]_{0}}$ | Relative active Cdk2. Used to fit EdU incorporation rates. |
| *Fit_CycE_T* | $\frac{[CycE]+[CycECdk2]+[CycECdk2\text{-}a]}{[CycE]_{0}+[CycECdk2]_{0}+[CycECdk2\text{-}a]_{0}}$ | Relative total CycE. Used to fit CycE. |

**References**

1. Li C, Donizelli M, Rodriguez N, Dharuri H, Endler L, Chelliah V, Li L, He E, Henry A, Stefan MI, Snoep JL, Hucka M, Le Novere N, et al. BioModels Database: An enhanced, curated and annotated resource for published quantitative kinetic models. BMC Syst Biol. 2010; 4:92.

2. Tyson JJ, Chen KC and Novak B. Sniffers, buzzers, toggles and blinkers: dynamics of regulatory and signaling pathways in the cell. Curr Opin Cell Biol. 2003; 15(2):221-231.

3. Goldbeter A and Koshland DE, Jr. An amplified sensitivity arising from covalent modification in biological systems. Proc Natl Acad Sci U S A. 1981; 78(11):6840-6844.
