# Supplementary material for: To senesce or not to senesce: how primary human fibroblasts decide their cell fate after DNA damage
Source: Aging (Albany NY). 2016 Jan 30;8(1):158–76. doi: 10.18632/aging.100883 (PMC4761720; doi:10.18632/aging.100883)
Supplement: Supplementary file 7 [file aging-08-158-s007.docx]

### Supplemental Figures





**Figure S5**: Time series of EdU incorporation, DNA content and senescence-associated β-galactosidase activity (SA-βG) after 10 Gy IR in MRC5 primary human fibroblasts. Left panel: EdU incorporation vs. DNA content; centre panel: DNA content with indicated percentages of G1, S and G2/M phase cells; left panel: DNA content vs. SA-βG. **A**) Control, **B**) 3 days after 10 Gy IR, **C**) 7 days after 10 Gy IR.


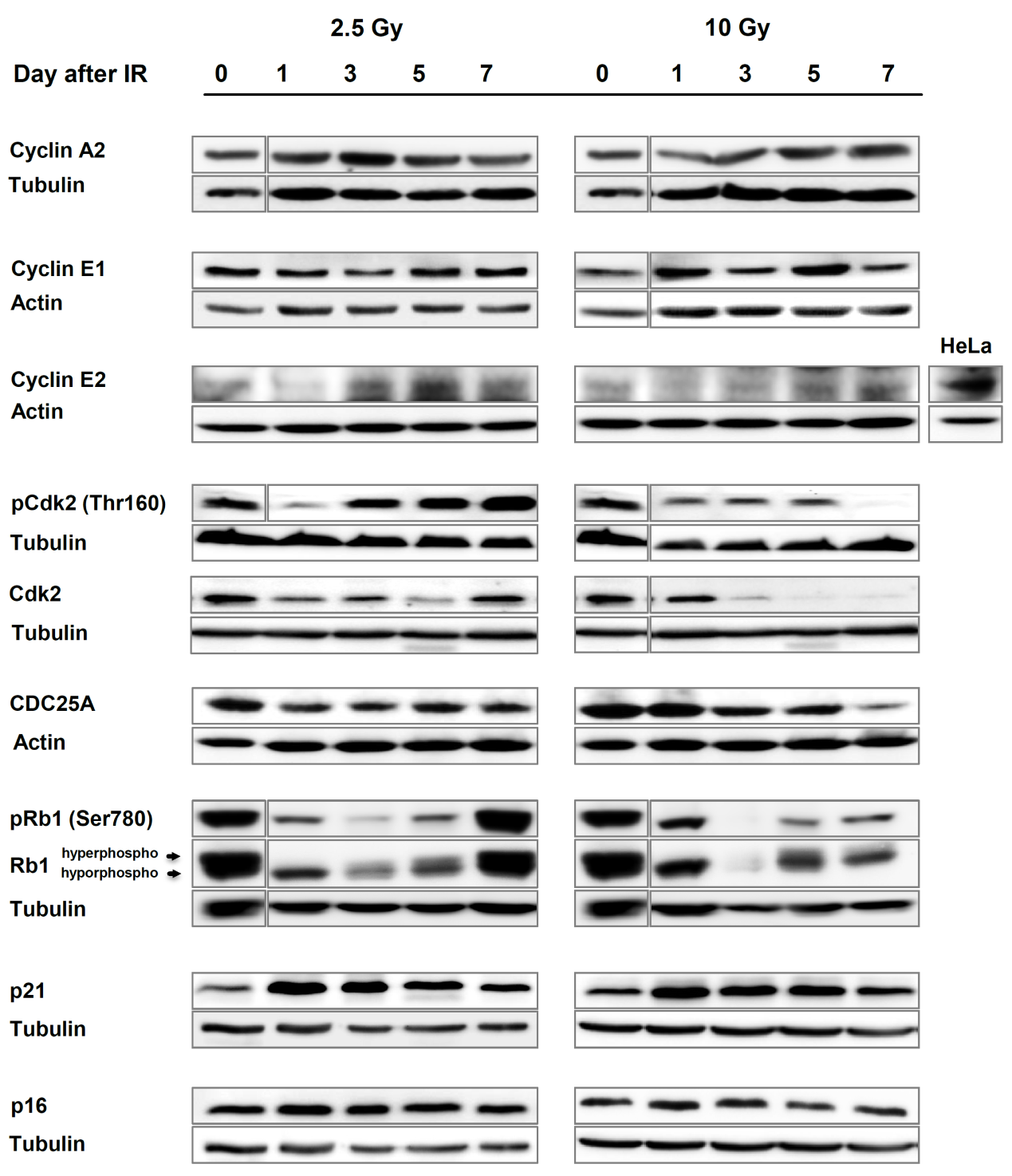


**Figure S6**: Representative Western Blots of irradiated MRC5 cells after recovery from 2.5 and 10 Gy IR for 0-7 days.





**Figure S7**: Histogram of switching points of the bistable region of *CycECdk2-a* steady states (Figure 4F) for the Monte-Carlo analysis, i.e. values of DDR corresponding to the minimum of the lower stable branch and the maximum of the upper stable branch, respectively. In case the number of γH2AX foci is lower than the minimum of the lower branch, the steady state switches to the upper stable branch, and if the number of γH2AX foci is higher than the maximum of the upper branch, the steady state switches to the lower stable branch.


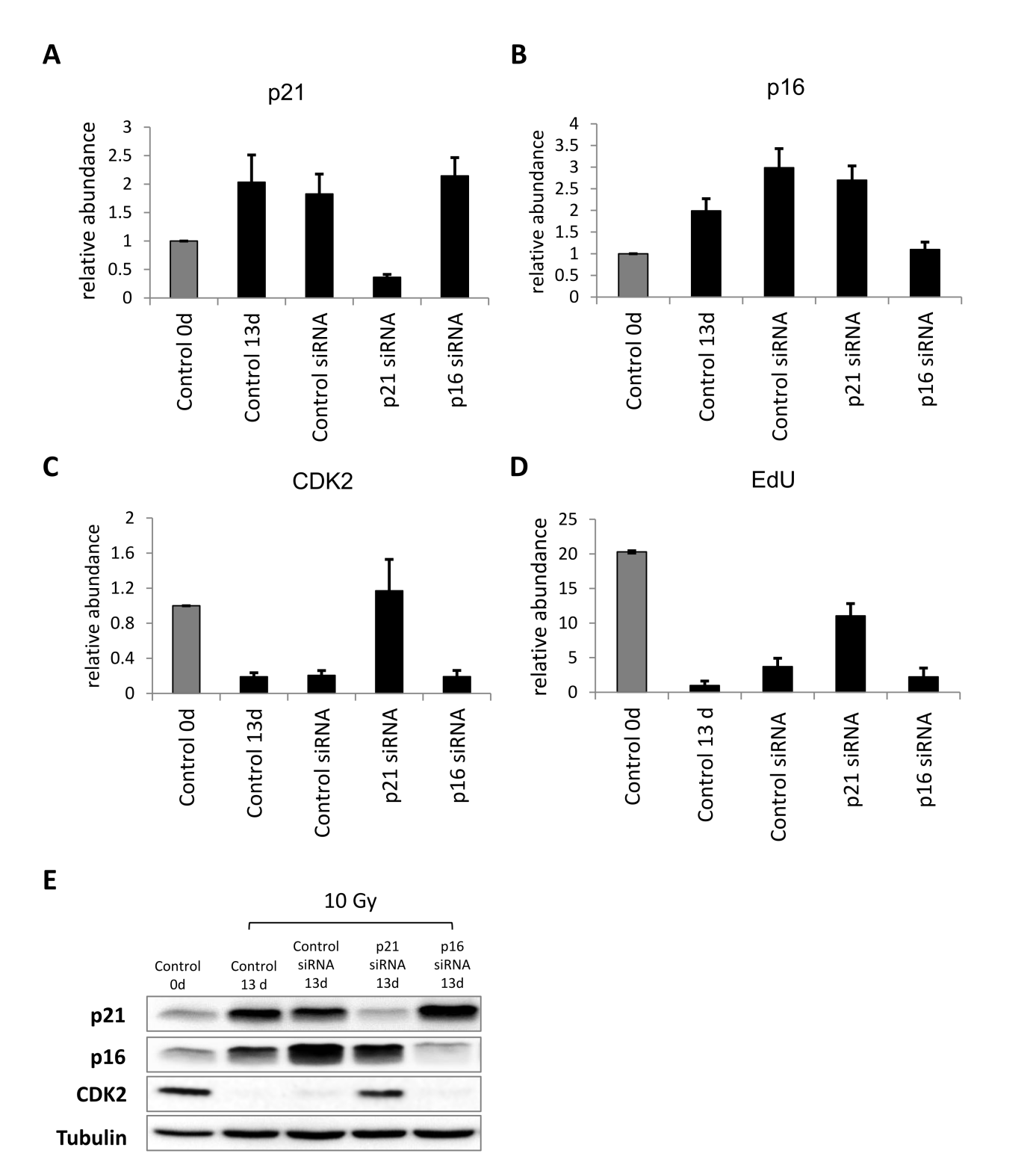


**Figure S8**: p21 and p16 silencing 11 days after IR with sample collection 2 days later (13d). **A**) relative p21 levels. **B**) relative p16 levels. **C**) relative Cdk2 levels. All values are scaled to day 0. **D**) relative EdU incorporation. Error bars indicate standard error of the mean (SEM) (n≥3). **E**) Representative western blot demonstrating validation of the p21 and p16 silencing in irradiated MRC5 cells (10 Gy).


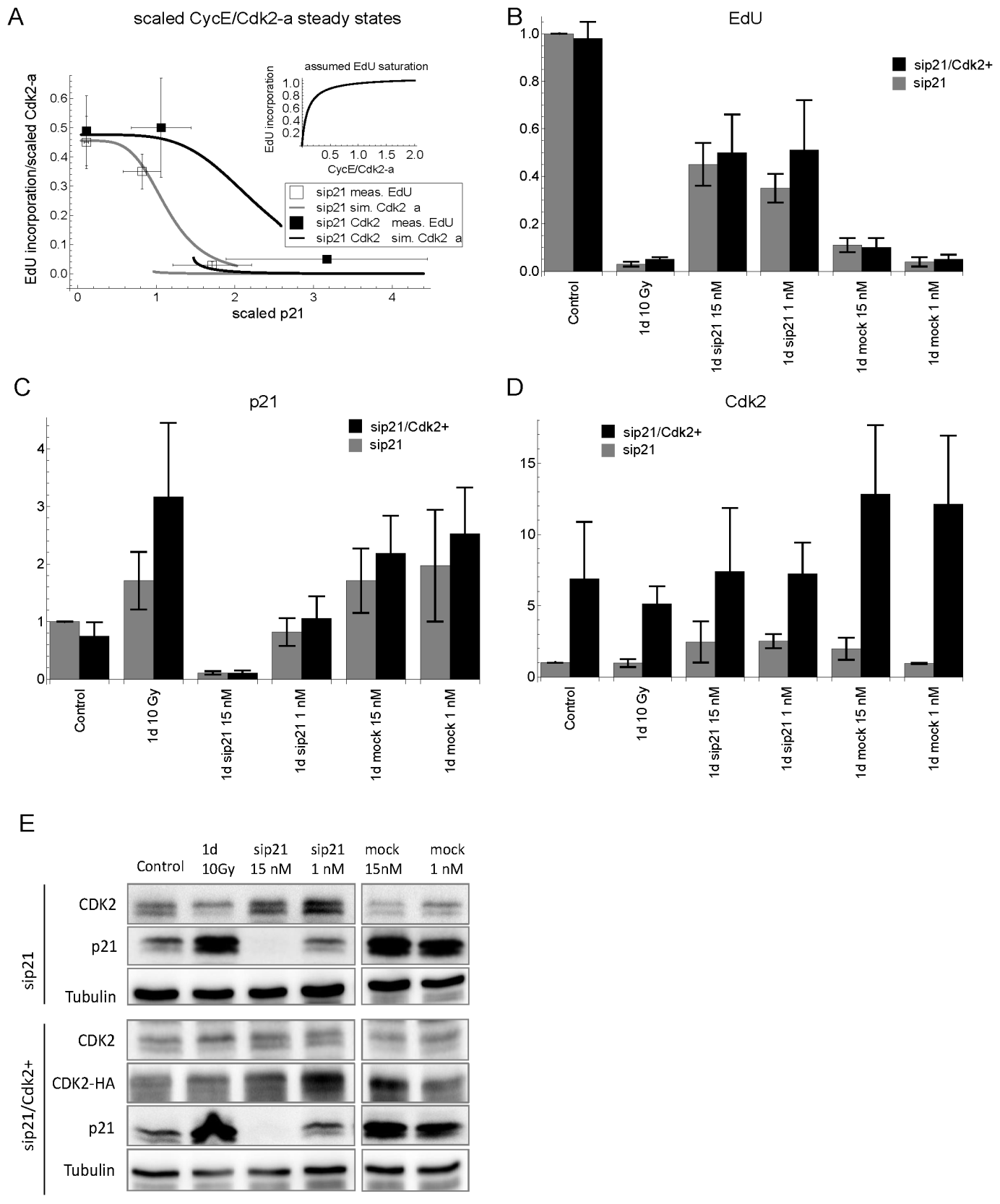


**Figure S9**: G1-S arrest 1 day after 10 Gy IR with p21 silencing and Cdk2 overexpression. **A**) Measured (mean ± SEM (n≥3)) and predicted EdU incorporation as a function of p21 abundance. Open squares: Measured EdU incorporation upon dose dependent p21 silencing (grey lines indicate according steady state simulations). Closed squares: Measured EdU incorporation upon dose dependent p21 silencing and concomitant Cdk2 overexpression (black lines indicate corresponding steady state simulations). Measured and simulated EdU incorporation are relative to control. The simulated steady state EdU levels where scaled to the measured EdU levels of the p21 silencing experiments (B) by a factor of 0.45, because we observed significantly lower EdU incorporation rates even when p21 was completely silenced (1d sip21 15nM). **Inset**: Here, we assumed that measured EdU incorporation are saturating with simulated *Cdk2-a* levels. We also scaled the simulated p21 levels to the measured ones (C) by a factor of 0.44. **B**) Measured EdU incorporation (mean ± SEM (n≥3)) after p21 silencing (sip21) and Cdk2 overexpression (Cdk2+). Values are scaled to the p21 silencing control experiment. **C**) Measured p21 (mean ± SEM (n≥3)) after p21 silencing (sip21) and Cdk2 overexpression (Cdk2+). Values are scaled to the p21 silencing control experiment. **D**) Measured Cdk2 (mean ± SEM (n≥3)) after p21 silencing (sip21) and Cdk2 overexpression (Cdk2+). Values are scaled to the p21 silencing control experiment. **E**) representative western blot demonstrating validation of the p21 silencing in control MRC5 cells and in MRC5 cells with Cdk2 overexpression.
