# Supplementary material for: To senesce or not to senesce: how primary human fibroblasts decide their cell fate after DNA damage
Source: Aging (Albany NY). 2016 Jan 30;8(1):158–76. doi: 10.18632/aging.100883 (PMC4761720; doi:10.18632/aging.100883)
Supplement: Supplementary file 8 [file aging-08-158-s008.docx]

### Supplemental Methods

***Immunofluorescent staining and image acquisition***

The immunofluorescent staining of cell nuclei and γH2AX foci was performed according to the following protocol. Cells grown on cover slips were washed in PBS and fixed in 4% paraformaldehyde (in 1xPBS, pH 7.4) for 15 min at room temperature. After three washing steps with PBS, cells were permeabilized using 0.1% Triton-X 100 (in 1xPBS, pH 7.4) for 15 min at room temperature and then incubated with the blocking reagent (5% Bovine serum albumin in 1xPBS, pH 7.4) for 45 min. The primary antibody anti-γH2AX (Ab26350, Abcam) was diluted to 1:1000 in 1% Bovine serum albumin, (in1xPBS pH 7.4) and added to the cells for 2 hours at room temperature. After the incubation, cells on cover slips were washed three times in PBS and the fluorescent-labelled secondary antibody (1:500) was added to the cells (IgG-Alexa488, Cell Sinaling #4408). The samples were stored in the dark at room temperature for 1 h. After washing, the DNA was stained with 49-6-diamidine-2-phenyl indole (DAPI, Invitrogen) diluted to a final concentration of 1μg/ml in the same buffer for 5 min at room temperature. Cells were then washed in PBS and 10 μl of antifade medium (Vectashield) was dropped onto clean slides and the cover slips were transferred onto the slides and fixed with nail polish. Then MRC5 cells were imaged using a confocal laser scanning microscope (FluoView1000, Olympus) with a 60×oil objective. For the quantification procedure of γH2AX foci, please refer to the Supplementary Material (Quantification of γH2AX foci time series).

***Overexpression of Cdk2 protein in MRC5 cells***

Lentiviral particles carrying Cdk2 gene were obtained by transfection of human embryonic kidney Lenti-X 293T cells (Clontech), seeded in 10 cm dishes (1.3-1.5 x 10^6^), using Lenti-Pac^TM^ HIV Expression Packaging Kit (GeneCopoeia) in combination with lentiviral expression vector containing either Cdk2-HA (pReceiver-Lv120) or GFP as a control (pReceiver-Lv105) (Genecopoeia). The following day (14h after transfection) media was replaced with fresh DMEM containing 5% FBS and 100 units/ml penicillin, 100 μg/ml streptomycin (Gibco). To infect primary human fibroblasts (MRC5), 1 x 10^6^ cells were seeded on 10 cm dish one day before the infection. The next day, cells were washed in PBS and infected with 10 ml of filtrated (0.45 µm membrane filter) viral supernatant in the presence of Polybrene (10 µg/ml). After 18 hours of incubation with lentivirus, fresh medium DMEM containing 10% FBS and 100 units/ml penicillin, 100 μg/ml streptomycin (Gibco) was added, and cells were seeded onto 6 cm dishes where they were subjected to p21 depletion followed by 10 Gy γ-irradiation.

***Dose-dependent p21 silencing in MRC5 cells with Cdk2 overexpression***

MRC5 cells overexpressing CDK2 or GFP protein were transfected using SignalSilence® p21 Waf1/Cip1 siRNA I (Cell signaling) targeting p21 protein or control siRNA (Cell signaling) in a final concentration of 15 nM or 1nM. siRNA transfection was performed using using RNAiMAX (Life Technologies) according to the manufacturer’s protocol.

Experimental scheme of RNAi in irradiated cells: MRC5 cells were transfected with p21 siRNA one day before they received 10 Gy IR dose and then were cultured for 24 hours. Afterwards, cells were processed for western blot and EdU incorporation procedures described above.

***Flow cytometry analysis of MRC5 with Cdk2-HA overexpression***

S-phase cells were pulse-labelled with EdU as described above (see EdU incorporation assay). EdU detection with the Click-iT EdU Flow cytometry assay kit (Alexa Fluor 647; Molecular probes) was performed according to the manufacturer's instructions. Cells overexpressing Cdk2-HA were fluorescently labelled by using primary antibody anti-HA (Thermo Scientific 26183; 1:500) and secondary antibody Alexa488 (Cell Sinaling #4408; 1:500). Analysis of EdU incorporation rate was performed only on cells which were positive for overexpressing Cdk2-HA. Flow cytometry was performed using the CyFlow space (Partec) and data was analysed using Flowing Software 2.5.1.

**References**

1. Debacq-Chainiaux F, Erusalimsky JD, Campisi J and Toussaint O. Protocols to detect senescence-associated beta-galactosidase (SA-betagal) activity, a biomarker of senescent cells in culture and in vivo. Nat Protoc. 2009; 4(12):1798-1806.

2. Noppe G, Dekker P, de Koning-Treurniet C, Blom J, van Heemst D, Dirks RW, Tanke HJ, Westendorp RG and Maier AB. Rapid flow cytometric method for measuring senescence associated beta-galactosidase activity in human fibroblasts. Cytometry A. 2009; 75(11):910-916.
